# Physicians’ views on the role of relatives in euthanasia and physician-assisted suicide decision-making: a mixed-methods study among physicians in the Netherlands

**DOI:** 10.1186/s12910-024-01031-1

**Published:** 2024-04-05

**Authors:** Sophie C. Renckens, Bregje D. Onwuteaka-Philipsen, Agnes van der Heide, H. Roeline Pasman

**Affiliations:** 1 Department of Public and Occupational Health, Amsterdam UMC, Location VU Medical Center, Amsterdam, The Netherlands; 2 Expertise Center for Palliative Care Amsterdam UMC, Amsterdam, The Netherlands; 3 Department of Public Health, Erasmus MC, University Medical Center Rotterdam, Rotterdam, the Netherlands

**Keywords:** Physician-assisted dying, Euthanasia, Family, End-of-life, Decision-making

## Abstract

**Background:**

Relatives have no formal position in the practice of euthanasia and physician-assisted suicide (EAS) according to Dutch legislation. However, research shows that physicians often involve relatives in EAS decision-making. It remains unclear why physicians do (not) want to involve relatives. Therefore, we examined how many physicians in the Netherlands involve relatives in EAS decision-making and explored reasons for (not) involving relatives and what involvement entails.

**Methods:**

In a mixed-methods study, 746 physicians (33% response rate) completed a questionnaire, and 20 were interviewed. The questionnaire included two statements on relatives’ involvement in EAS decision-making. Descriptive statistics were used, and multivariable logistic regression analyses to explore characteristics associated with involving relatives. In subsequent interviews, we explored physicians’ views on involving relatives in EAS decision-making. Interviews were thematically analysed.

**Results:**

The majority of physicians want to know relatives’ opinions about an EAS request (80%); a smaller group also takes these opinions into account in EAS decision-making (35%). Physicians who had ever received an explicit EAS request were more likely to want to know opinions and clinical specialists and elderly care physicians were more likely to take these opinions into account. In interviews, physicians mentioned several reasons for involving relatives: e.g. to give relatives space and help them in their acceptance, to tailor support, to be able to perform EAS in harmony, and to mediate in case of conflicting views. Furthermore, physicians explained that relatives’ opinions can influence the decision-making process but cannot be a decisive factor. If relatives oppose the EAS request, physicians find the process more difficult and try to mediate between patients and relatives by investigating relatives’ objections and providing appropriate information. Reasons for not taking relatives’ opinions into account include not wanting to undermine patient autonomy and protecting relatives from a potential burdensome decision.

**Conclusions:**

Although physicians know that relatives have no formal role, involving relatives in EAS decision-making is common practice in the Netherlands. Physicians consider this important as relatives need to continue with their lives and may need bereavement support. Additionally, physicians want to perform EAS in harmony with everyone involved. However, relatives’ opinions are not decisive.

**Supplementary Information:**

The online version contains supplementary material available at 10.1186/s12910-024-01031-1.

## Background

In 2022, 76% of physicians in the Netherlands had ever received an explicit request for euthanasia or physician-assisted suicide (EAS) [1]. Euthanasia is defined as the intentional termination of a patient’s life at their explicit request by a physician who administers lethal medication. In physician-assisted suicide the patient self-administers the lethal medication prescribed by a physician. In the Netherlands, EAS is allowed if physicians adhere to the legal due care criteria [2]. When physicians receive an EAS request, they must consider whether they want to grant that request. Physicians are not obliged to perform EAS and can weigh their own considerations when deciding on an EAS request. Many different considerations play a role when physicians decide on an EAS request. These considerations can be related to the interpretation and perception of the legal criteria (e.g. is the physician satisfied that the request is well-considered?), but physicians may also base their decision on considerations that have little or nothing to do with the legal criteria (e.g. a physician considers it important that patients accept their situation and are at peace with it in order to perform EAS) [3].

EAS decision-making has typically been framed in the patient-physician dyad. However, recently it has been suggested that a triad model in which relatives also play a role seems to be more appropriate for describing what goes on in EAS practice [4, 5]. The role of relatives in EAS (decision-making) is not addressed in the Dutch euthanasia law [2]. Over the years, the Dutch Royal Medical Association (KNMG) has published several position papers on the EAS practice. In their first position paper in 1984 EAS was framed within the patient-physician relationship and relatives were not mentioned [6]. Subsequently in 1995 and 2003, the KNMG described that, although EAS is in principle a matter between an individual patient and a physician, relatives are typically closely involved [7, 8]. It is emphasized in the position papers that considering doctor-patient confidentiality, a patient's wish not to discuss an EAS request with relatives should be respected. Furthermore, the KNMG mentioned that the opinion of relatives is not decisive, yet physicians are advised to investigate possible objections and try to overcome them as much as possible. The most recent position paper, published in 2021, adds to this that physicians can include the opinion of relatives in their considerations [9]. However, it is not explained what “including the opinion of relatives into consideration” should mean in practice, e.g. to what extent is it advised to take into account relatives’ opinion?

Previous research shows that once a patient requests EAS, a process of deliberation, decision-making and ultimately performance starts, during which it is not unusual that relatives are involved [3, 5, 10, 11]. In 2021, 57% of the physicians who had performed EAS discussed it with relatives during the EAS process [1]. This number suggests that a substantial share of physicians at least discuss the EAS (request) with relatives, but it is unknown whether these physicians also want to know what the relatives think about the EAS request. Conflicting views between patients and relatives about an EAS request can complicate the EAS process according to physicians [5, 10]. Although multiple studies have shed some light on the position of relatives in EAS decision-making, it remains unclear how common it is for physicians to want to involve relatives, and especially why they feel that relatives should or should not be closely involved. Therefore, we examined how many physicians in the Netherlands involve relatives in EAS decision-making and explored the reasons why they do or do not involve relatives and what involvement entails.

## Methods

### Design

As part of the fourth evaluation of the Dutch euthanasia act [1], we conducted a sequential explanatory mixed-methods study among physicians in the Netherlands. We started with a retrospective cross-sectional questionnaire, which was followed by in-depth semi-structured interviews. The questionnaire results informed the development of the interview topic guide, and the interviews provided a deeper understanding of certain quantitative findings. Furthermore, the questionnaire results were used to purposively sample physicians for the interviews, as well as to personalise, to some extent, the individual interviews.

### Data collection and study population

#### Questionnaire

A written invitation to participate in an online questionnaire study was sent to the home or work addresses of a random sample of 1100 general practitioners, 1000 clinical specialists (working in hospitals) and 400 elderly care physicians (stratified by specialty). We obtained addresses from a national database of registered physicians (IQVIA). Eligible participants were physicians who had worked in adult patient care in the Netherlands in the past year. Invitations included a personal log-in code and a link to the online questionnaire. If physicians logged-in and consented to participate, they got access to a separate webpage with the questionnaire. Thereby anonymity was ensured without precluding the possibility of sending two reminders to non-responders. The last reminder included an abbreviated 2-page questionnaire on paper. Quantitative data were collected between April and September 2022.

Of the 2500 invited physicians, 245 did not meet the inclusion criteria. Of the 2255 eligible physicians, 746 responded (33%). Among the non-responders (*n* = 1509), 39 provided a reason for non-response, with lack of time (*n* = 28) being the most frequently reported reason, followed by having no (recent) experience with EAS (*n* = 7), and four other reasons.

#### Interviews

At the end of the questionnaire, we asked physicians if they consented to be contacted for a follow-up interview. A total of 81 physicians provided consent. To ensure diversity, purposive sampling was used to select the interview participants. Variation was sought with regard to specialty, gender, working experience, and perceived pressure of patients and their relatives on EAS requests. Primarily physicians with experience with EAS requests were selected so that they could speak from their own experience. Interviews were conducted in two sequential rounds. In the first round (September–October 2022), 22 physicians were contacted for an interview, 15 of whom agreed to participate. The seven physicians who did not participate were unavailable by phone and/or email or had time constraints. Of the 15 interviews, 11 addressed the topic of the current study: the role of relatives in EAS decision-making. Due to time constraints the role of relatives in EAS decision-making was not discussed in four other interviews. The latter interviews covered other topics that were relevant for the fourth evaluation of the Dutch euthanasia act (e.g. preferences for euthanasia versus physician-assisted suicide). Six of the 11 interviews were conducted by SCR and the other five interviews by two other researchers. All interviewers had no prior relation to the participating physicians. The average duration of the interviews was 37 min (ranging from 24 to 60 min). After analysing the interviews from the first round, we concluded that not all themes regarding the role of relatives in EAS decision-making were sufficiently explored**.** For example, we did not fully understand yet what the main reasons were for not taking relatives’ opinions into account and to what extent the opinion of relatives was a decisive factor in the decision-making process. Therefore, a second round of interviews with other physicians, from the 81 that signed up, was conducted (August–September 2023). Of the 14 physicians who were contacted, nine participated. The five physicians who did not participate, could not be reached by phone and/or email. All interviews were conducted by SCR. In contrast to the first round of interviews which also covered other topics related to EAS, the interviews in the second round focused fully on the role of relatives in EAS decision-making, and were therefore shorter. On average, these interviews took 14 min (ranging from 11 to 20 min). All 20 interviews were conducted by telephone or video call, based on the physicians’ preference. All interviews were audio-recorded and transcribed verbatim.

### Measurements

#### Questionnaire

The questionnaire used in this study was similar to the questionnaire used in the previous three evaluations of the euthanasia act [12–14] (Additional file 1). It covered a wide range of questions regarding physicians’ experiences and views on EAS. For the purposes of the current study, we focused on two items related to physicians’ views on the role of relatives in decision-making regarding EAS requests: 1) I want to know close relatives’ opinions about an EAS request, and 2) I take close relatives’ opinions into account in my decision about an EAS request. Additionally, we used data from the questionnaire on the demographic and professional characteristics of the physicians, including medical specialty (general practitioner/ clinical specialist/elderly care physician), work experience (in years), age (in years), gender (male/ female/ other), religion (yes/ no), being a palliative care consultant/member palliative care team (yes/ no) and experience with explicit EAS requests (never received an explicit EAS request/ ever received an explicit EAS request, but never performed EAS/ ever performed EAS).

#### Interviews

The interviews were semi-structured and used a topic list. The topic list in the first round of interviews included multiple topics, including physicians’ views on the role of relatives in EAS decision-making (Additional file 2). For the current study, we focus on the data on this topic. In the second round of interviews, this was the only topic that was addressed and therefore discussed more elaborately. The topic was introduced by referring to the interviewees’ answers to the questionnaire regarding the involvement of relatives in EAS decision-making (e.g. “In the questionnaire you indicated that you would like to know relatives’ opinions about an EAS request, but do not take this into account in your decision to grant a request or not. Could you explain a bit more about this?). Next, the interviewer explored in more detail reasons for (not) wanting to know close relatives’ opinions and for (not) taking these opinions into account. Furthermore, questions were asked concerning discordant opinions about an EAS request between patients and relatives.

### Analysis

#### Questionnaire

Statistical analyses were performed using SBSS IBM 28. For each specialty, a weight factor was calculated in order to make the sample representative of all physicians in the Netherlands working in the included specialties. Descriptive statistics were used to assess background characteristics using the weight factors, for both the total population and for three specialties separately. The two items on the role of relatives in decision-making were also analysed using descriptive statistics. In the analyses on the statement about taking relatives’ opinions into account, only physicians who indicated that they wanted to know relatives’ opinions were included. To explore which variables were associated with wanting to know relatives’ opinions and with taking relatives’ opinions into account in EAS decision-making, univariable and multivariable logistic regression analyses were conducted. These analyses were done separately for the two statements. Independent variables that were tested for associations were gender, age, religious belief, speciality, work experience, being a palliative care consultant/member palliative care team, being a SCEN physician, and experience with explicit EAS requests. In the logistic regression analyses of the second statement, the agree category was compared to the disagree category, and the neutral category was not included. Additionally, the agree category was compared to the neutral category; the results of these analyses are reported in Additional file 3. All variables that had a *p*-value less than 0.10 in univariable logistic regression analyses were included in multivariable logistic regression analysis, in which a *p*-value < 0.05 was considered statistically significant.

#### Interviews

The interviews were transcribed verbatim and analysed following the principles of thematic analysis [15]. First, SCR familiarised herself with the data from the eleven interviews of the first round by reading the transcripts thoroughly. Next, interviews were coded inductively by SCR using MAXQDA 2020. These codes were extensively discussed with HRP and BOP, which resulted in refinement of some codes. The interviews were then again coded by SCR using the refined codebook. After analysis of the first round of interviews, we concluded that additional interviews were needed. The interviews from the second round were coded using the codebook of the first round. This codebook was then refined based on the new data from the second round. This resulted in a final codebook, with which the interviews from the first round were also coded. During coding 20 interviews based on this final codebook, no new themes emerged from the data and therefore we concluded that no additional interviews were needed. Finally, the quotes were translated by a professional translator and checked by a second professional translator.

## Results

The physicians’ background characteristics are shown in Table 1. The majority of physicians were female (54.3%), they had a median age of 48 years and 28.9% were religious. Physicians had a median work experience of 20 years, 5.3% were palliative care consultants and/or members of a palliative care team and 2.3% were SCEN physicians. Just over half of the physicians (54.7%) had ever performed EAS. Of the 20 interviewed physicians, 10 were general practitioners, six were elderly care physicians, and four were clinical specialists (Table 2). Furthermore, 11 interviewed physicians were male, they had 24 years of working experience on average, and all but one had experience with performing EAS.

**Table 1:** Background characteristics of physicians from the questionnaire (*n* = 746)*

	**Total** Total *n* = 746 N (%)
**DEMOGRAPHICS**	
**Gender**	
Male	328 (45.6%)
Female	416 (54.3%)
Other	1 (0.1%)
**Age (years)**	
Median (IQR)	48.0 (16.0)
**Religious belief**^**a**^	
Yes	241 (28.9%)
**PROFESSIONALS CHARACTERISTICS**	
**Medical specialty**	
General practitioner	402 (57.9%)
Clinical specialist	196 (36.3%)
Elderly care physician	144 (5.8%)
**Years of working experience**	
Median (IQR)	20.0 (16.0)
**Consultant palliative care/ member palliative care team**	
Yes	43 (5.3%)
**SCEN physician**^**b**^	
Yes	23 (2.3%)
**Ever received an explicit EAS request**	
No	169 (25.0%)
Yes, but never performed EAS	165 (20.3%)
Yes, and ever performed EAS	410 (54.7%)

^*^Weighted percentages. As a result of this weighting procedure the percentages presented cannot be derived from the absolute unweighted numbers presented

^a^According to the respondent, Christian religion in 89.6% of the cases

^b^A SCEN physician is a trained physician from whom other physicians can obtain information and advice about EAS, or request a formal consultation (one of the criteria for due care)

Missing values: gender 1, age 4, religious belief 5, medical specialty 4, years of working experience 5, consultant palliative care/member of palliative care team 4, SCEN physician 4, ever received an explicit EAS request 2

**Table 2:** Characteristics of relatives participating in interviews

	**Medical specialty**	**Gender**	**Work experience**	**Wants to know relatives’ opinion**	**Takes into account relatives’ opinion**
1	GP*	Male	21–30 years	Agree	Neutral
2	ECP*	Male	> 30 years	Agree	Disagree
3	GP	Female	21–30 years	Agree	Disagree
4	Clinical specialist	Male	6–10 years	Agree	Agree
5	GP	Male	11–20 years	Agree	Disagree
6	GP	Female	> 30 years	Agree	Disagree
7	GP	Male	21–30 years	Agree	Disagree
8	Clinical specialist	Male	> 30 years	Agree	Agree
9	ECP	Female	6–10 years	Agree	Disagree
10	ECP	Female	> 30 years	Agree	Neutral
11	ECP	Female	11–20 years	Agree	Disagree
12	ECP	Male	> 30 years	Agree	Disagree
13	GP	Female	21–30 years	Agree	Agree
14	GP	Female	21–30 years	Agree	Disagree
15	GP	Male	21–30 years	Neutral	Disagree
16	GP	Female	11–20 years	Agree	Disagree
17	Clinical specialist	Male	> 30 years	Agree	Agree
18	ECP	Female	21–30 years	Agree	Agree
19	Clinical specialist	Male	> 30 years	Agree	Agree
20	GP	Male	≤ 5 years	Disagree	Disagree

* *GP* General practitioner, *ECP *Elderly care physician

### Views on involving relatives in EAS decision-making

**Fig. 1:**
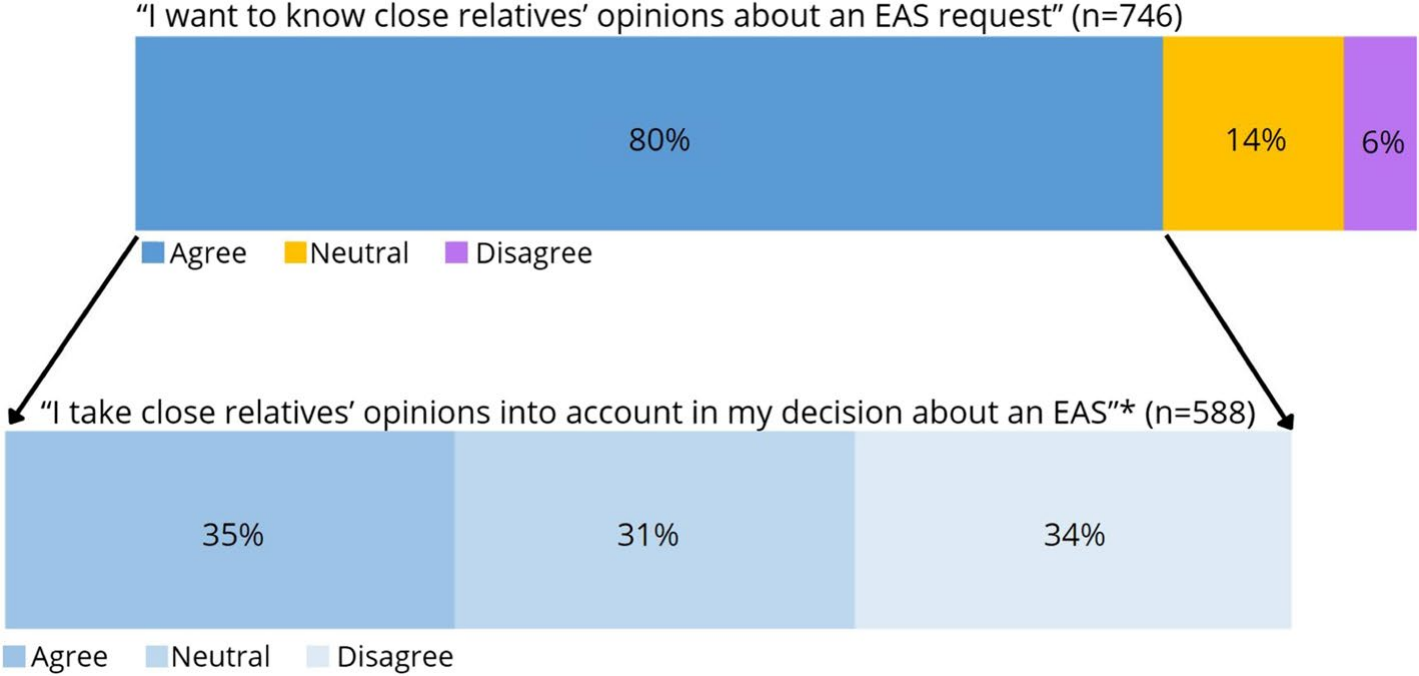
Proportion (weighted percentages) of physicians who want to know close relatives’ opinions about an EAS request and take these opinions into account in their EAS decision-making (*n* = 746). * Includes data of the 80% (*n* = 588) of physicians that indicated that they want to know close relatives’ opinions. Missing values: first item 8, second item 2

The large majority of physicians (80%) indicated that they wanted to know close relatives’ opinions about an EAS request of their loved one (Fig. 1). Fourteen percent of the physicians did not agree or disagree with the statement that they want to know close relatives’ opinions and 6% reported that they do not want to know. Physicians who indicated that they want to know the opinions of close relatives were divided on whether they include these opinions in their decision-making on the EAS request. Approximately one third (35%) of these physicians indicated that they take close relatives’ opinions into account, another one third (34%) that they do not, and the last one third (31%) was neutral about it.

### Determinants of wanting to know close relatives’ opinions in EAS decision-making

The multivariable logistic regression showed that physicians who had ever received an explicit EAS request were more likely to want to know close relatives’ opinions compared to physicians who had never received an explicit EAS request (Table 3). This was the case both for physicians who had received an explicit request but never performed EAS (OR 1.98), as for physicians who also had performed EAS (OR 1.83).

**Table 3:** Association between physicians’ demographics and professional characteristics and wanting to know close relatives’ opinions in EAS decision-making (row % and ORs; agree vs. rest) (*n* = 746)

	**Agree**	**Univariable**	**Multivariable**
	Row %	OR (95% CI)	OR (95% CI)
**DEMOGRAPHICS**			
**Gender**^**a**^			
Male (*n* = 324)	77.2%	1.00	
Female (*n* = 412)	81.6%	1.31 (0.91–1.88)	
**Age (years)**^**b**^		0.99 (0.97–1.01)	
**Religious belief**			
No (*n* = 268)	79.4%	1.00	
Yes (*n* = 131)	80.0%	0.96 (0.66–1.42)	
**PROFESSIONAL CHARACTERISTICS**			
**Specialty**			
General practitioner (*n* = 218)	80.4%	1.00	
Clinical specialist (*n* = 103)	76.7%	0.80 (0.53–1.22)	
Elderly care physician (*n* = 78)	81.8%	1.10 (0.67–1.79)	
**Years of working experience**^**b**^		1.00 (0.98–1.02)	
**Consultant palliative care/member palliative care team**			
No (*n* = 375)	79.3%	1.00	
Yes (*n* = 25)	86.0%	1.61 (0.67–3.89)	
**SCEN physician**			
No (*n* = 385)	79.7%	1.00	
Yes (*n* = 15)	78.3%	0.91 (0.33–2.50)	
**Ever received an explicit EAS request**			
No (*n* = 76)	71.1%	1.00	1.00
Yes, but never performed EAS (*n* = 89)	82.9%	**1.98 (1.17–3.45)**	**1.98 (1.17–3.45)**
Yes, and ever performed EAS (*n* = 237)	81.8%	**1.83 (1.20–2.78)**	**1.83 (1.20–2.78)**

^a^There is one physician who indicated to have gender ‘other’. This is treated as a missing value in this analyses due to problems with statistical power

^b^Continuous variable, therefore no row percentage shown

Missing values: gender 2, age 3, religious belief 3, specialty 3, years of working experience 1, consultant palliative care/member of palliative care team 2, SCEN physician 2

### Determinants of including relatives’ opinions in EAS decision-making

The multivariable model showed that among physicians who want to know close relatives’ opinions about an EAS request, clinical specialists and elderly care physicians were more likely to take these opinions into account compared to GPs (respectively OR 3.36 and OR 1.91) (Table 4). Other demographic and professional characteristics were not found to be statistically significant associated with including relatives’ opinions in EAS decision-making in the multivariable analyses. Differences between the agree and neutral categories are described in Additional file 3, which shows similar but smaller differences than in the agree versus disagree analysis.

**Table 4:** Association between physicians’ demographics and professional characteristics and taking into account close relatives’ opinions in their decision-making or not (row % and ORs; agree vs. disagree) (*n* = 402)

	**Agree**	**Univariable**	**Multivariable**
	Row %	OR (95% CI)	OR (95% CI)
**DEMOGRAPHICS**			
**Gender**^**a**^			
Male (*n* = 167)	52.1%	1.00	-
Female (*n* = 233)	48.9%	0.88 (0.59–1.31)	-
**Age (years)**^**b**^		1.01 (0.99–1.03)	**-**
** Religious belief**			
No (*n* = 268)	50.7%	1.00	**-**
Yes (*n* = 131)	48.1%	0.90 (0.59–1.37)	**-**
**PROFESSIONAL CHARACTERISTICS**			
**Specialty**			
General practitioner (*n* = 218)	39.0%	1.00	1.00
Clinical specialist (*n* = 103)	68.9%	**3.47 (2.11–5.71)**	**3.36 (1.93–5.85)**
Elderly care physician (*n* = 78)	56.4%	**2.03 (1.20–3.42)**	**1.91 (1.11–3.26)**
**Years of working experience**^**b**^		1.02 (1.00–1.04)	-
**Consultant palliative care/member palliative care team**			
No (*n* = 375)	50.4%	1.00	-
Yes (*n* = 25)	52.0%	1.07 (0.47–2.40)	-
**SCEN physician**			
No (*n* = 385)	50.6%	1.00	-
Yes (*n* = 15)	46.7%	0.85 (0.30–2.40)	-
**Ever received an explicit EAS request**			
No (*n* = 76)	60.5%	1.00	1.00
Yes, but never performed EAS (*n* = 89)	56.2%	0.84 (0.45–1.56)	1.22 (0.62–2.43)
Yes, and ever performed EAS (*n* = 237)	44.7%	**0.53 (0.31–0.89)**	0.93 (0.50–1.70)

^a^There is one physician who indicated to have gender ‘other’. This is treated as a missing value in this analyses due to problems with statistical power

^b^Continuous variable, therefore no row percentage shown

Missing values: gender 2, age 3, religious belief 3, specialty 3, years of working experience 1, consultant palliative care/member of palliative care team 2, SCEN physician 2

### Reasons for (not) wanting to know relatives’ opinions

Among the 20 physicians interviewed, one physician stated in the questionnaire that he did not want to know relatives’ opinions about an EAS request. However, during the interview this physician realised that, contrary to what he reported in the questionnaire, it is important for him to give relatives space to share their thoughts and that he does ask relatives how they feel about their loved one’s EAS request:

*“I noticed it took some thinking about... At first, I was quite insistent in thinking that didn’t have a role at all, but I gradually realised that – in practice at any rate – I do provide an opportunity to express that.”* (20: general practitioner, male, ≤5 years of working experience)

In addition to this physician, one physician was neutral about wanting to know relatives’ opinions about an EAS request, while all other interviewed physicians wanted to know this information. In the interviews, physicians provided several reasons for this. One of the reasons given by multiple physicians for wanting to know what relatives think about an EAS request is to give them space to share their feelings, as they need to move on with their lives after the death of their loved one. Physicians felt, partly based on experience, that sharing and discussing these feelings helps relatives to accept the death of their loved one and the way in which their loved one died. As one physician explained:

“*I often want to hear from the relatives about what they know or what they think, especially how they feel about their relative’s request, as that’s often how it happens. Sometimes you get a lot of anger, which can happen of course. Along the lines of ‘why are you giving up now?’ Bringing that out into the open does often lead to more acceptance. But it doesn’t influence my opinion. I do think it’s a good idea to discuss it, though, because of that acceptance*.” (5: general practitioner, male, 11-20 years of working experience)

Physicians described that knowing how relatives feel about their loved one’s EAS request can help them provide tailored support before, during and after the performance of the EAS. For example, relatives may have difficulty coping with their loved one’s decision and their imminent death, which may in turn result in complicated grief:

“*Well, it’s more so that you can support the family too because it’s an emotional process, it’s a tough process, not just for the patient but also for the family. So they deserve some support. Right, because otherwise there will be problems with the mourning process.*” (12: elderly care physician, male, >30 years of working experience)

“*If they have problems with it, you can offer support. For instance, can you expect a delayed mourning process or a depression? And might this cause them problems?*” (16: general practitioner, female, 11-20 years of working experience)

A few physicians mentioned that they had experienced that patients did not want to tell their relatives about the upcoming EAS. These physicians indicated that they thought relatives must always know about the EAS request of their loved one and did not want to perform EAS if relatives are not aware:

*“I once had a patient who wanted a kind of secret euthanasia. He said: I’m not going to say goodbye to anyone. I said I wouldn’t allow that. Because those people have to be able to move on, I said. I’m not saying you have to see every single one of them, but I do think you should give the most important people the chance to say something to you if they want*.” (13: general practitioner, female, 21-30 years of working experience)

In addition, some physicians experienced that knowing what relatives think about an EAS request can reveal a lack of practical information about EAS procedures. Physicians can provide support accordingly if they are aware of this:

“*It’s more the fear, the hesitation, just the practical stuff, that they simply don’t know what to expect. Some people think it takes a really long time, others that it’s really quick. You can really focus on the procedure, as I find there’s a lot that needs to be explained.*” (20: general practitioner, male, ≤5 years of working experience)

It is also possible that relatives do not agree with their loved one’s decision. Several interviewed physicians indicated that they aim to carry out EAS in harmony. Therefore, being aware of conflicting views, gives them the opportunity to mediate:

“*You know, it’s also about the person making this decision, the patient, because they won’t have a particularly pleasant death if they know the children are sitting there thinking ‘Dad, this thing you’re doing, it’s not what I want’. It’s also important for the person who will be passing away to know that it will take place in harmony, or reasonable harmony, that they can leave things behind in a good state.*” (16: general practitioner, female, 11-20 years of working experience)

“*I can imagine that if the patient wants it and the family are against it, that you still try to mediate between them. Without changing the patient’s mind but trying to get some sort of understanding so you can have a decent farewell without it being unbearable for father or mother*.” (9: elderly care physician, female, 6-10 years of working experience)

In addition, some physicians said that they do not want to have any tension in the last phase of a patient’s life, particularly at the moment of performing EAS. If they know that relatives have difficulties with EAS, they can discuss this first. They want to avoid tension for the patient, the relatives or themselves:

I: “*What’s the most important reason for wanting to know how the relatives feel about it?*”

R: “*Um… well, because you don’t really want rows when you go, or during someone’s final stage. Right, this is really an important time for saying goodbye. And if all that leads to is arguments between relatives, well, you wouldn’t wish that on anyone.”*

*I: “Who don’t you want that for?*”

R: “*Not for the patient and not for their partner, but not for the others either. And also not for me, because it’s not pleasant working with a family that has a lot of issues.*” (13: general practitioner, female, 21-30 years of working experience)

Several physicians also indicated that they want to know relatives’ opinions because as physicians they had to continue working with the relatives. For example, some physicians were also the treating physician of the relatives. They stated that they do not want a conflict after performing EAS:

“*So I find it really important for the family to agree about this. In part because I don’t fancy having some kind of conflict. You just know the family will turn up afterwards, and right, I simply don’t want to deal with all that grumbling.*” (4: clinical specialist, male, 6-10 years of working experience)

One physician also indicated that the fear of prosecution played a role in this:

“*I do think it’s a good idea to have everyone on the same page. That everyone understands it at any rate. And well, there’s a bit of self-interest here, as you’ll have officially committed a criminal act when you’ve performed euthanasia. You do still have... I can’t bear the thought that I might perform euthanasia and then get taken to court because a relative didn’t agree with it.*” (3: general practitioner, female, 21-30 years of working experience)

Finally physicians explained that information about how relatives feel about an EAS request provides valuable information for them to determine whether they feel that all due care criteria can be met. Information from relatives can provide insight into whether the request is well-considered, and whether the suffering is unbearable and without prospect of improvement:

“*The family’s viewpoint can help, because the euthanasia legislation talks of empathizing with the suffering. Sometimes the family can clarify that a bit, so I get more of a feeling for it. If they tell me their father was always an energetic man for whom nothing was ever too much and he, well... and if they tell me they’ve seen their father deteriorate so much, or their mother has deteriorated so much, and they no longer get the impression their father or mother enjoys life, that it has become very hard for them, the relatives can make that suffering, well, make it easier to empathize with.*” (18: elderly care physician, female, 21-30 years of working experience)

I: “*Why do you find it so important to know what the relatives think of it?*”

R: “*To get confirmation of what that person was like during their life. You know, because usually the family will say, ‘This is really typical of the patient. He always used to say that too.’ I often see that as confirmation. And if the request is completely unexpected, that’s something you need to reflect on. [...] And also to take the collateral history: what was someone’s approach to life and are you surprised by the request, or do you think it’s absolutely typical…? Well, I do find that very important.*” (11: elderly care physician, female, 11-20 years of working experience)

### Reasons for (not) taking into account relatives’ opinion

Although a substantial part of physicians (35%) indicated in the questionnaire that they consider relatives’ opinions when deciding whether to grant an EAS request, none of them stated in the interviews that this opinion is actually a decisive factor. According to some interviewed physicians, the opinion of relatives does influence the process of granting the EAS request and carrying out the EAS. However, lack of support from relatives does not ultimately prevent these physicians from granting the EAS request. Yet, when relatives do not support their loved one’s decision, physicians find it more difficult to perform EAS.

R: “*I think it would be very difficult to continue with euthanasia if the family don’t agree with it or can’t understand it. I don’t think that would be nice at all. I’d do everything I can to make sure they understand it. [...]*”

I: “*Would that actually stop you from granting the request, do you think?*”

R: “*No, I don’t think that’s possible. If the patient actually says to me, ‘I don’t have any quality of life, I’ve got unbearable suffering with no prospect of improvement, I’m asking you as my doctor for euthanasia’, I can’t say ‘I’m not doing it because your child doesn’t want it’. I can’t say that because I’d be leaving my patient in the lurch.*” (17: clinical specialist, male, >30 years of working experience)

R: “*Well, to the extent that I think you should do your level best to create harmony. […] If that doesn’t work out — again, I’ve never had that happen — then I think, in the end, you should discuss it with the patient because they’re the person you want to be accountable to, so you discuss with them how you’re going to approach it and who will be present and who won’t. So yes, it does make it more complex, but my responsibility is to the patient and not to the family or relatives. So that’s how you should approach it then.*”

I: “*So it doesn’t necessarily have to be an obstacle to carrying it out*.”

R: “*Not in the end. But it does make it more complex.*” (19: clinical specialist, male, >30 years of working experience)

There were also several physicians who were very clear in the interviews that they do not take the views of relatives into account when making decisions about EAS. The main reason for this was that, according to the interviewed physicians, one of the core principles of EAS is patient autonomy. The patient is the only person who can decide whether to request EAS and the request must stem from a patient’s wish, not from relatives. Relatives may have a vested interest in supporting or opposing their loved one’s EAS request. Thus, the decision to grant that request is a process between the physician and the patient.

“*It really needs to be kept separate… someone is dying and they’re the only one who is dying, so they’re the only person who has a say. […] I find that the person who gets euthanasia, or undergoes it or however you want to phrase it, I think that’s the person I have a direct patient relationship with. So together we are the ones taking the decision. Normally, you’d do that anyway without the relatives involved and they don’t really have a say at all. In this kind of case too, I still think that applies. [...] If your loved one is dying, you often want to hold on to them for as long as possible. Not necessarily, for sure, but I imagine people feeling that way, so they might have an interest in us not doing it. Right, that’s quite understandable. And sometimes they might have an interest in us actually doing it. If there’s a big inheritance coming and it’s done quickly and you want to sell that house, well, that’s quite convenient. But you should just block out such potential interferences, it has to be an unbiased decision. So all these other things shouldn’t be considered at all. So that’s a reason for keeping it as clean a process as possible.*” (14, general practitioner, female, 21-30 years of working experience)

“*Strictly speaking, it’s the patient who makes the euthanasia request. They can give their independent opinion about this. And I go along with that. Whether the relatives agree or disagree with this doesn’t interest me. What matters to me is what the patient thinks.*” (15: general practitioner, male, 21-30 years of working experience)

One physician explained that she was very certain about not taking the views of relatives into account when deciding whether to grant an EAS request, not only because of her responsibility to her patient, but also to protect relatives from having to take on a potentially burdensome role. According to this physician, having to make decisions about the life of a loved one is an impossible and undesirable task:

“*I think you need to keep them out of it for their own good too, to make it easier for them. Otherwise they’ll think they have some influence and they might end up feeling guilty, if you don’t watch out. So I think it’s important for them too, protecting them from a role in the decision-making, because they basically don’t have one. Not just formally, but not in practice either. It’s also much better for them not to have to take that decision because I reckon it’s very difficult for the family to have to decide whether or not your loved one is allowed to die. I don’t think you can do that. So really you have to... they certainly have a voice and of course they can help think and talk about how and what and when — that’s fine. But the decision about whether or not to do it just isn’t theirs to take. And I think it would be really horrible for them too.*” (14: general practitioner, female, 21-30 years of working experience)

Several physicians said that they always make it clear to relatives that they have no say in the EAS decision-making process, to avoid misunderstandings or false expectations:

“*I always tell the family, ‘It’s nice that we can have this chat. I want to know what you think about it all, and your feelings. But you need to realise that if you don’t agree to it, that won’t mean I’m not going to carry it out. But I do want us to end up on the same page. So I do tell them that, but I make it clear they don’t have a veto and I don’t need their consent.*” (11: elderly care physician, female, 11-20 years of working experience)

### Dealing with conflicting views of patient and relatives on an EAS request

Some physicians indicated that the first step in dealing with conflicting views of patients and relatives is to find out what the relatives’ objections are and what they are based on. For example, this could be based on religious beliefs, personal values and norms, or fear of losing a partner:

“*I want to know the issue in particular. For example, if they have conscientious objections due to their religion, what’s behind it? So I can take that into account in my explanation. I try to see it from their perspective.*” (20: general practitioner, male, ≤5 years of working experience)

“*I think then I’d start a conversation with them and keep at it, asking them if this is going to stay a firm no, is there something we can do about it, can I still...Try to find out why it’s a no. There can be all kinds of reasons for that.*” (16: general practitioner, female, 11-20 years of working experience)

Physicians explained that when they know why relatives are opposed to their loved one’s EAS request, they organise a meeting with the relatives in which they try to increase their understanding of the decision by providing them with information about the reasons for the request and procedures. Some physicians experienced that objections are based on misunderstandings that can be clarified through discussion.

“*Right, then you start talking to make it clearer. Because often it’s... if the family can’t understand or accept something, it’s because... well, they don’t know enough, there’s too little information. So you explain and talk to them and try to get them to the point where they say ‘OK, now I understand it with this new information’.*” (17: clinical specialist, male, >30 years of working experience)

“*Right, try to probe and ask what’s the problem, what makes it so difficult. Often there’s something underlying this. A fear or a negative experience. They might say, my mother-in-law had a death like this and I found it really horrible because it went very weirdly or whatever... You know, then you might be able to remove some of their unease or concerns by explaining this is what can happen. So really what I already said, my experience is that if you engage the relatives – the future surviving relatives – properly in the whole process then it can often be something really special.*” (16: general practitioner, female, 11-20 years of working experience)

In addition to talking to relatives themselves, some physicians said that they encouraged patients to discuss their EAS request further with their relatives.

“*Yes, or I let the family do it [talk about the difference of opinion] themselves, because of course the patient is still in a position to do that themselves, and of course that works best if they can explain why they are making a particular choice*.” (13: general practitioner, female, 21-30 years of working experience)

“*That task [getting the relatives to agree] is also for the person who is dying. They are that person’s relatives so that person has work to do, you could say. To explain it to them.*” (14: general practitioner, female, 21-30 years of working experience)

Finally, one physician explained that he also wants to know from patients how they feel about their loved one not supporting their EAS request:

“*It is also possible that the family doesn’t agree with the euthanasia request. So then I talk to the patient again, ask if they heard that and understood it. What do you want to do about it? Will this affect your opinion or your request? In that way, I try and bring the family and patient together after all.*” (12: elderly care physician, male, >30 years of working experience)

## Discussion

Relatives are important in the EAS decision-making practice according to physicians in the Netherlands. The majority of physicians want to know what close relatives think about the EAS request of their loved one. Physicians who had ever received an explicit EAS request were more likely to want to know opinions compared to physicians who had never received an explicit EAS request. Several reasons were provided for wanting to know relatives’ opinions. First, physicians want to give relatives space and help them accept the way in which their loved one dies. They may also provide tailored support, both practical and emotional. Second, physicians want to perform EAS in a harmonious manner, minimising tension and preventing conflicts. Finally, knowing relatives’ views provides opportunities for mediation in the case of conflicting views and helps physicians evaluate whether they think the due care criteria can be met. Physicians’ views on whether to take relatives’opinion into account in EAS decision-making are diverse. Approximately one third of the physicians indicated taking close relatives’ opinions into account, one third was neutral, and one third reported not taking it into account. Clinical specialists and elderly care physicians were more likely to take these opinions into account compared to GPs. Physicians explained that relatives’ opinions can influence the process of making a decision about an EAS request and eventually about performing EAS but should never be a decisive factor. If relatives are not supportive of the EAS request, physicians find the process more difficult. Physicians who do not take relatives’ opinions into account explained that patient autonomy should not be undermined and that EAS is a decision between a physician and patient. They also argue that relatives should be protected from feeling that they have a say in the decision about their loved one’s life. In cases where patients and relatives have conflicting views on an EAS request, physicians try to get them on the same page by investigating relatives’ objections and providing appropriate information.

### Despite having no legal position in EAS, relatives play a role in practice

Considering that relatives have no legal position in the practice of EAS in the Netherlands, one-third of physicians taking relatives’ opinions into account when deciding whether to grant an EAS request may seem high. However, the interviews clarified that taking relatives’ opinions into account does not mean that this opinion is a decisive factor for physicians. Although lack of support from relatives ultimately does not withhold physicians in our study from performing EAS, they do find it more difficult to perform EAS in these cases. Several other qualitative studies also reported that opposition from relatives and unresolved conflicts can impede the decision-making process. However, in contrast to what we found these studies found that some physicians did not grant an EAS request if they felt that relatives could not cope with it, or if there was a conflict [3, 5, 11]. Van Zwol et al. (2022) did not describe the latter as reasons for refusing an EAS request, but did find that physicians reported that relatives who do not support a request require attention and time [10]. If relatives had difficulty with accepting the imminent death of the patient, physicians tried to get them on board [10]. Similarly, physicians in our study explained that their goal was to perform EAS in a harmonious way, and they therefore put effort in trying to get relatives support their loved one’s EAS request.

Since the majority of physicians want to know what relatives think of an EAS request, some of them may have to deal with relatives who do not support the request. It can be valuable for physicians who have little experience with EAS decision-making, and particularly with dealing with conflicting views, to learn from more experienced physicians, e.g. on if and how a physician should have a mediating role. Similar information could be included in the KNMG position paper to give physicians guidance in dealing with conflicting views.

### Why are certain physicians more inclined to involve relatives in EAS decision-making than others?

Two professional characteristics are associated with involving relatives in the EAS decision-making process. First, physicians who have ever received an explicit EAS request are more likely to want to know close relatives’ opinions about an EAS request. It is possible that physicians with experience with EAS requests are more aware that in practice EAS decision-making and performance take place in a social system, and that most patients are not isolated individuals. Second, compared with GPs, clinical specialists and elderly care physicians are more likely to take relatives’ opinions into account when making EAS decisions. Although it is not entirely clear why this is the case, we can think of two possible explanations for it. First, compared with GPs, clinical specialists and elderly care physicians tend to have shorter treatment relationships with their patients and less experience with EAS requests: they may therefore rely more on relatives’ opinions to substantiate their decision. Whereas GPs usually know their patients longer than elderly care physicians and clinical specialists, they are likely to better know the physical, psychological and social context. Therefore they may feel more confident to make a well-considered decision about an EAS request and rely less on relatives. Second, it is more common for elderly care physicians and clinical specialists to involve relatives in their daily clinical practice, as relatives often accompany the patient during appointments, whereas GPs tend to have more one-on-one contact with their patients. Although the latter might change during the last phase of life, GPs are likely to be more used to making decisions with the patient only. This may explain why elderly care physicians and clinical specialists are more likely to involve relatives in EAS decision-making.

### Professional position papers compared to EAS decision-making in practice

Our finding that physicians consider relatives’ opinions when deciding about an EAS request aligns with the most recent position paper of the KNMG [9]. As the KNMG did not further explain what including the opinion of relatives into considerations means, our study provides valuable insights. The KNMG position papers over time show a shift from the traditional patient-physician dyad toward a triad model in in which patients, physicians and relatives play a role in EAS decision-making, by increasingly paying attention to the role of relatives in these papers over the years [6–9]. This finding is in accordance with several studies that proposed a triad model as a more accurate representation of the EAS decision-making in practice [4, 5]. The views and experiences of physicians in our study support the triad model to some extent, namely involvement of relatives in the EAS trajectory and communication, but no say in the actual decision-making. The majority of physicians involve relatives in EAS decision-making, although to various extents. They consider it valuable to involve relatives for the relatives themselves (e.g. to be able to provide tailored support), for the physicians (e.g. to obtain extra insight for the due care criteria) and for patients (e.g. to try to reconcile and perform EAS in harmony in case of conflicting views).

### International relevance

To our knowledge, nearly all studies that provided some insight into the role of relatives in EAS decision-making were conducted in the Netherlands. However, EAS is legal in an increasing number of countries and several countries are crafting or considering EAS legislation [16]. Also in these countries, patients probably have closely involved relatives with whom physicians need to deal in some way during EAS decision-making. Therefore, our study also provides valuable insights for other countries, which can be considered when reviewing or crafting EAS legislation.

### Strength and limitations

One of the strengths of this study is the mixed-methods design. The interview data helped to explain certain quantitative findings, which might have been interpreted differently if there would not have been qualitative findings. An example of this is that based on the questionnaire data one might think that for one third of the physicians relatives’ opinion might play a very big or even decisive role when deciding about an EAS request, while the interviews revealed a more nuanced practice. Furthermore, this is one of the few studies with quantitative data on this topic.

A limitation of this study is the relatively low response rate (33%). In addition, the perspective of physicians who do not want to know relatives’ opinions about an EAS request is lacking, because there was only one physician of this group available for an interview who during the interview turned out to have a preference for actually knowing relatives’ opinions. Therefore, we cannot provide further insight into why physicians do not want to know relatives’ opinions. Further inquiry into this perspective is needed. Furthermore, we only included the views of physicians in this study although it concerns the interaction between physicians, patients and relatives. We recommend future research to include the perspectives of patients and their close relatives. Such studies could look into what role patients see for their relatives in EAS decision-making and their preferences in this regard, how relatives experience their role in the decision-making, how many relatives support the decision of their loved one, whether they feel they were involved in the decision-making process and whether the level of involvement is associated with complicated grief.

## Conclusions

In conclusion, involving relatives in EAS decision-making is common practice for physicians in the Netherlands. Physicians feel that it is important to consider relatives’ opinions as relatives need to continue with their lives, relatives might need additional support, and physicians want to perform EAS in harmony for everyone who is involved. However, involving relatives does not mean that relatives have a say in the ultimate decision about EAS. As many physicians will have to deal with relatives in EAS decision-making and potentially with conflicting views, it would be valuable to provide more elaborate information on the role of relatives in EAS decision-making in the professional position papers of the KNMG.

### Supplementary Information

**Supplementary Material 1.**

**Supplementary Material 2.**

**Supplementary Material 3.**

## Data Availability

The dataset used and/or analysed during the current study is available from the corresponding author upon reasonable request.

## Abbreviations

EAS: Euthanasia and physician-assisted suicide
KNMG: Dutch Royal Medical Association

## References

[1] van der Heide A, Legemaate J, Onwuteaka-Philipsen B, Bosma F, van Delden H, Mevis P (2023). Vierde evaluatie Wet toetsing levensbeëindiging op verzoek en hulp bij zelfdoding [Fourth evaluation of the Termination of Life on Request and Assisted Suicide Act].

[2] Termination of life on request and assisted suicide (Review procedures) Act, 194. 2001. 10.2143/ep.9.2.503855 15712446

[3] Ten Cate K, van Tol DG, van de Vathorst S (2017). Considerations on requests for euthanasia or assisted suicide; a qualitative study with Dutch general practitioners. Fam Pract.

[4] Roest B, Trappenburg M, Leget C (2019). The involvement of family in the Dutch practice of euthanasia and physician assisted suicide: a systematic mixed studies review. BMC Med Ethics.

[5] Snijdewind MC, van Tol DG, Onwuteaka-Philipsen BD, Willems DL (2014). Complexities in euthanasia or physician-assisted suicide as perceived by Dutch physicians and patients' relatives. J Pain Symptom Manage.

[6] Koninklijke Nederlandse Maatschappij tot bevordering der Geneeskunst [Duth Royal Medical Association]. Standpunt inzake euthanasie [Position on euthanasia]. 1984.

[7] Koninklijke Nederlandse Maatschappij tot bevordering der Geneeskunst [Duth Royal Medical Association]. Standpunt Hoofdbestuur 1995 inzake euthanasie [Position 1995 on euthanasia]. 1995.

[8] Koninklijke Nederlandse Maatschappij tot bevordering der Geneeskunst [Duth Royal Medical Association]. Standpunt Federatiebestuur KNMG inzake euthanasie 2003 [Position Federal Board KNMG on euthanasia 2003]. 2003.

[9] Koninklijke Nederlandse Maatschappij tot bevordering der Geneeskunst [Duth Royal Medical Association]. KNMG standpunt Beslissingen rond het levenseinde [KNMG position paper End of life decisions]. 2021.

[10] van Zwol M, de Boer F, Evans N, Widdershoven G (2022). Moral values of Dutch physicians in relation to requests for euthanasia: a qualitative study. BMC Med Ethics.

[11] Dees MK, Vernooij-Dassen MJ, Dekkers WJ, Elwyn G, Vissers KC, van Weel C (2013). Perspectives of decision-making in requests for euthanasia: a qualitative research among patients, relatives and treating physicians in the Netherlands. Palliat Med.

[12] Onwuteaka-Philipsen B, van der Heide A, Legemaate J, van Delden H, Evenblij K, El Hammoud I (2017). Derde evaluatie Wet toetsing levensbeëindiging op verzoek en hulp bij zelfdoding [Third evaluation of the termination of life on request and assisted suicide act].

[13] van der Heide A, Legemaate J, Onwuteaka-Philipsen B, Bolt B, Bolt I, van Delden H (2012). Tweede evaluatie Wet toetsing levensbeëindiging op verzoek en hulp bij zelfdoding [Second evaluation of the Termination of Life on Request and Assisted Suicide Act].

[14] Onwuteaka-Philipsen BD, Gevers JKM, van der Heide A, van Delden JJM, Pasman HRW, Rietjes JAC (2007). Evaluatie Wet toetsing levensbeëindiging op verzoek en hulp bij zelfdoding [Evaluation of the Termination of Life on Request and Assisted Suicide Act].

[15] Braun V, Clarke V (2006). Using thematic analysis in psychology. Qual Res Psychol.

[16] Mroz S, Dierickx S, Deliens L, Cohen J, Chambaere K (2020). Assisted dying around the world: a status quaestionis. Ann Palliat Med..

