# Supplementary material for: Physicians’ views on the role of relatives in euthanasia and physician-assisted suicide decision-making: a mixed-methods study among physicians in the Netherlands
Source: BMC Med Ethics. 2024 Apr 5;25:43. doi: 10.1186/s12910-024-01031-1 (PMC10996154; doi:10.1186/s12910-024-01031-1)
Supplement: Supplementary file 1 — Supplementary Material 1. [file 12910_2024_1031_MOESM1_ESM.docx]

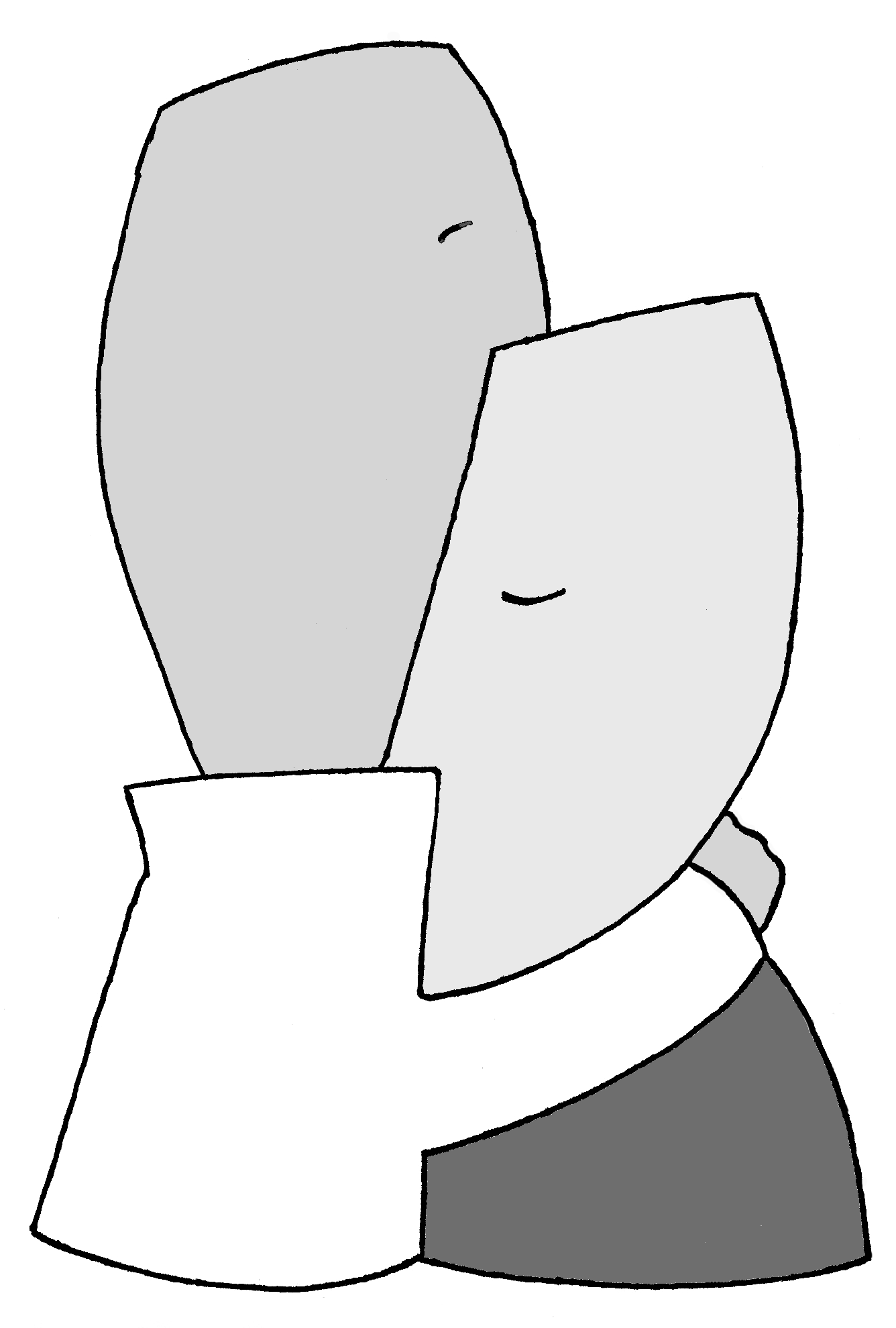


Evaluation Termination of Life at Request

and Assisted Suicide Review Act [translated]

National study into euthanasia and physician-assisted suicide

**Instruction**

**You will remain anonymous when completing this questionnaire. Logging in with the personal login code is separate from completing the substantive questionnaire.**

Most questions only require you to select **one box**. If a question allows you to select **more than one box**, this is clearly stated.

You can save your answers in between by clicking on the "save in between" button at the top right of the page. Completing the questionnaire will take around 20-30 minutes depending on your experiences with euthanasia and physician-assisted suicide.

When asked about your experiences in the past year, the **12 months** prior to completing this questionnaire
are meant.

It is important to use the following definitions when answering the questions:

**Euthanasia**: Intentional termination of a patient’s life at their explicit request by a physician who administers lethal medication.

**Physician-assisted suicide**: Intentional termination of a patient’s life at their explicit request with the patient self-administering the lethal medication prescribed by a physician.

**Note:** In the questionnaire, we refer to euthanasia for brevity's sake. This refers to **both euthanasia and physician-assisted suicide**.

If you have any questions or comments following this survey or questionnaire, please contact:

| **A. Firstly** | | | |
| --- | --- | --- | --- |
|  | Have you worked as a physician in patient care in the past year? | - Yes | |
|  |  | - No 🡪 You do not need to complete this questionnaire further. Thank you very much for your interest. Please remember to send your answer by clicking on 'send answers'. | |
|  | What is your specialty? | - General practitioner | |
|  |  | - Elderly care physician | |
|  |  | - Internist | |
|  |  | - Cardiologist | |
|  |  | - Surgeron | |
|  |  | - Pulmonologist | |
|  |  | - Neurologist | |
|  |  | - Intensivist | |
|  |  | - Other, **namely:** | |
|  | 1. How many years have you been working as a physician? | year | |
|  | 1. How many hours per week do you work as a physician? | hours per week | |
|  | Do you work as a palliative care consultant or SCEN physician?  *(one or more answers possible)* | - No | |
|  |  | - Yes, SCEN physician | |
|  |  | - Yes, palliative care consultant | |
|  |  | - Yes, part of a palliative team | |
| **B. Your experiences with euthanasia** | | | |
|  | 1. Has a patient ever asked you for euthanasia **in due time** (where the patient does not yet want it in the short term, but, for example, when the suffering would become too bad)? | - Yes | |
|  |  | - No 🡪 forwarded to **question B2** | |
|  | 1. **If yes,** how many patients made such a request **in due time in the past year**? | patients | |
|  |  | - None | |
|  | 1. Has a patient ever **explicitly asked** you for euthanasia in **the foreseeable future** (where the patient wants euthanasia in the short term)? | - Yes | |
|  |  | - No 🡪 forwarded to **question B4** | |
|  | 1. How many patients made an **explicit request** for euthanasia in the **foreseeable future** to you **in the past year**? | patients | |
|  |  | - None | |
|  | 1. Have you ever **performed euthanasia** (at the patient's explicit request)? | - Yes | |
|  |  | - No 🡪 forwarded to **question B3d** | |
|  | 1. In how many patients have you **performed** euthanasia **in the past year**? | patients | |
|  |  | - No | |
|  | 1. How many of these patients did assist with suicide (physician-assisted suicide)? | Patient | |
|  |  | - No | |
|  | 1. Where did these explicit requests (questions B2b & B3b) for euthanasia mainly stem from **in the past year**? *(If someone falls into several categories, please choose the most applicable one)*   **Suffering due to:** | Number of requests received | Number of requests granted |
|  | cancer |  |  |
|  | another life-threatening physical condition |  |  |
|  | dementia |  |  |
|  | a psychiatric disorder |  |  |
|  | an accumulation of health problems related to old age |  |  |
|  | there was no serious physical or psychiatric condition (completed life) |  |  |
|  | 1. How many cases of euthanasia have you reported to an euthanasia review committee **in the past year**? | cases | |
|  | 1. Is it **conceivable** that you will perform euthanasia in the future? | - Yes 🡪 forwarded to **question B5** | |
|  |  | - No | |
|  | 1. **If no**, would you refer a patient with a euthanasia request to another physician (who might be willing to perform it)? | - Yes | |
|  |  | - No | |
|  | 1. Do you think euthanasia should be prohibited? | - Yes | |
|  |  | - No | |

| In the questionnaire below, we ask you to indicate whether you have ever acted as in the situations described, or whether it would be conceivable that you will ever act this way. | | | | |  |
| --- | --- | --- | --- | --- | --- |
|  | **Did you ever perform euthanasia on a patient…** | | | |  |
|  |  | **Yes** | **No,** but **conceivable** | **No, inconceivable** |  |
|  | 1. due to suffering from cancer? |  |  |  |  |
|  | 1. due to suffering from another life-threatening physical condition? |  |  |  |  |
|  | 1. due to suffering from **early-stage** dementia, where the patient still had decisional capacity? |  |  |  |  |
|  | 1. due to suffering from **advanced** dementia, based on a written euthanasia declaration, where there was an additional serious condition? |  |  |  |  |
|  | 1. due to suffering from **advanced** dementia, based on a written euthanasia declaration, where there was **no** **additional serious condition**? |  |  |  |  |
|  | 1. due to suffering from a psychiatric condition? |  |  |  |  |
|  | 1. due to suffering from an accumulation of health problems related to old age |  |  |  |  |
|  | 1. due to suffering through 'completed life'/'tired of living' with a medical basis for the suffering, **without** any physical or psychiatric condition? |  |  |  |  |
|  | 1. who was **younger** than 18 years |  |  |  |  |
|  |  |  |  |  | |
|  | **Has it occured that…** | **Yes** | **No,** but **conceivable** | **No, inconceivable** |  |
|  | 1. you kept a patient in deep sedation or coma continuously until death? |  |  |  | |
|  | 1. you prescribed medication to a patient for the purpose of **enabling** the patient to **end life** at any time **(without the** presence of a doctor)? |  |  |  | |
|  | 1. you administered a drug for the purpose of terminating life **without** the patient having expressly requested it |  |  |  | |
|  | 1. you supported a patient, who deliberately stopped eating and drinking with the aim of dying, during his final weeks? |  |  |  | |
|  | 1. you, in such a patient who had consciously stopped eating and drinking, administered palliative sedation? |  |  |  | |
|  | 1. you gave a patient with a wish for euthanasia the **idea of** dying by stopping eating and drinking? |  |  |  | |
|  | 1. you refused a euthanasia request **because** you thought palliative sedation was a better option? |  |  |  | |
|  | 1. you pointed out the possibility of euthanasia to a patient on your own initiative? |  |  |  | |
|  | Has there been any occasion when a patient of yours died as a result of consciously stopping eating and drinking for the purpose of dying? (**not** in the context of anorexia in a terminal illness) | - Yes | | | |
|  |  | - No | | | |

| **C. Requests and performance** | | | | | | | | | | | | | | | | | | | | | | | | |
| --- | --- | --- | --- | --- | --- | --- | --- | --- | --- | --- | --- | --- | --- | --- | --- | --- | --- | --- | --- | --- | --- | --- | --- | --- |
|  | 1. Have you **ever refused** a request for euthanasia? (This **does not** include cases where the patient withdrew the request himself or the patient died before euthanasia could be performed) | | | | | | | | | | | - Yes | | | | | | | | | | | | |
|  |  |  |  |  |  |  |  |  |  |  |  | - No 🡪 forwarded to **question C3** | | | | | | | | | | | | |
| b | 1. **If yes**, how many times in the past year have you refused a request for euthanasia? | | | | | | | | | | | times | | | | | | | | | | | | |
|  | 1. Have you **ever** refused a request for euthanasia in a situation where due care criteria were met but you had personal objections? | | | | | | | | | | | - Yes | | | | | | | | | | | | |
|  |  |  |  |  |  |  |  |  |  |  |  | - No 🡪 forwarded to **question C3** | | | | | | | | | | | | |
|  | 1. **If yes,** what were your personal objections? | | | | | | | | | | |  | | | | | | | | | | | | |
|  |  | | | | | | | | | | | | | | | | | | | | | | | |
|  | In all cases where you refuse a euthanasia request, would you refer the patient to another physician*?*  *(one or more answers possible)* | | - Yes, I will always refer | | | | | | | | | | | | | | | | | | | | | |
|  |  |  | - No, I will **not** refer if: | | | | | | | | | | | | | | | | | | | | | |
|  |  |  | - - The patient's condition is too bad | | | | | | | | | | | | | | | | | | | | | |
|  |  |  | - - I am certain that the due care criteria have not been met | | | | | | | | | | | | | | | | | | | | | |
|  |  |  | - - I can adequately support the patient in other ways | | | | | | | | | | | | | | | | | | | | | |
|  |  |  | - - Different situation, **namely**: | | | | | | | | | | | | | | | | | | | | | |
|  |  |  | - No, I will never refer | | | | | | | | | | | | | | | | | | | | | |
|  | 1. Have you ever referred a patient to the Euthanasia Expertise Centre? | | - Yes | | | | | | | | | | | | | | | | | | | | | |
|  |  |  | - No, but it is **conceivable** that I will one day 🡪 forwarded to **question C4c** | | | | | | | | | | | | | | | | | | | | | |
|  |  |  | - No, I will **never** do this 🡪 forwarded to **question C5** | | | | | | | | | | | | | | | | | | | | | |
|  | 1. In what situation(s) have you **referred** to the Euthanasia Expertise Centre?   *(one or more answers possible)* | | - Life-threatening condition | | | | | | | - Accumulation of health problems related to old age | | | | | | | | | | | | | | |
|  |  |  | - Dementia | | | | | | | - Tired of living | | | | | | | | | | | | | | |
|  |  |  | - Psychiatric suffering | | | | | | | - Other, **namely:** | | | | | | | | | | | | | | |
|  | 1. In what situation(s) would you consider referral to the Euthanasia Expertise Centre **conceivable**?   *(one or more answers possible)* | | - Life-threatening condition | | | | | | | - Accumulation of health problems related to old age | | | | | | | | | | | | | | |
|  |  |  | - Dementia | | | | | | | - Tired of living | | | | | | | | | | | | | | |
|  |  |  | - Psychiatric suffering | | | | | | | - Other, **namely:** | | | | | | | | | | | | | | |
|  | In the past **5 years,** have you had the following experiences? | | | | | | | | | | | | | | | | | | | | Yes | | | No |
|  | 1. I was under the impression that a patient had made the euthanasia request under **pressure from relatives** | | | | | | | | | | | | | | | | | | | |  | | |  |
|  | 1. I felt pressured **by a patient** to decide on euthanasia | | | | | | | | | | | | | | | | | | | |  | | |  |
|  | 1. I felt pressured **by relatives** of a patient to decide on euthanasia | | | | | | | | | | | | | | | | | | | |  | | |  |
|  | 1. I felt pressured **by relatives** of a patient to **refuse** the euthanasia request | | | | | | | | | | | | | | | | | | | |  | | |  |
|  | 1. I felt pressured by a patient or their relatives to perform euthanasia **as soon as possible** | | | | | | | | | | | | | | | | | | | |  | | |  |
|  | 1. I felt pressured **by colleagues** to **refuse** the request | | | | | | | | | | | | | | | | | | | |  | | |  |
|  | 1. I felt pressured **by colleagues** to decide on euthanasia | | | | | | | | | | | | | | | | | | | |  | | |  |
|  | 1. I felt pressured **by management to refuse** the request | | | | | | | | | | | | | | | | | | | |  | | |  |
|  | 1. I felt pressured **by management** to decide to euthanise | | | | | | | | | | | | | | | | | | | |  | | |  |
|  | 1. In the past **five years,** have you **ever not reported** a case of euthanasia to a review committe? | | | | | | | | | | - Yes | | | | | | | | | | | | | |
|  |  |  |  |  |  |  |  |  |  |  | - No 🡪 forwarded to **question C7** | | | | | | | | | | | | | |
|  | 1. **If yes**, in the most recent case, what was the reason for **not** reporting? | | | | | | | | | | | | | | | | | | | | | | | |
|  |  | | | | | | | | | | | | | | | | | | | | | | | |
|  | 1. Is it **conceivable** that you might **not** report a case of euthanasia in the future? | | | | | | | | | | - Yes | | | | | | | | | | | | | |
|  |  |  |  |  |  |  |  |  |  |  | - Not conceivable 🡪 forwarded to **Section D** | | | | | | | | | | | | | |
|  | 1. **If yes**, in what situation(s) would you not report a case of euthanasia? | | | | | | | | | | | | | | | | | | | | | | | |
|  |  | | | | | | | | | | | | | | | | | | | | | | | |
| **D. Last request for euthanasia or physician-assisted suicide** | | | | | | | | | | | | | | | | | | | | | | | | |
| In the past 5 years, have you ever had explicit requests for euthanasia from... (*one or more answers possible)*   - A patient with dementia - A patient with an accumulation of health problems related to old age - Another patient - I have not had an explicit request in the last 5 years 🡪 forwarded to Section E | | | | | | | | | | | | | | | | | | | | | | | | |
|  | | **If dementia,** this latest explicit request concerned a patient with dementia.. | | | | - who made a verbal request | | | | | | | | | | | | | | | | | | |
|  | |  |  |  |  | - about whom those involved indicated that the patient, now without decisional capacity, was at that time in the situation described in the euthanasia declaration | | | | | | | | | | | | | | | | | | |
|  | |  |  |  |  | - Neither🡪 forwarded to **Section E** | | | | | | | | | | | | | | | | | | |
|  | | What did you do with this last explicit request? | | | | - I **granted** the request **and performed euthanasia** 🡪 forwarded to question D4 | | | | | | | | | | | | | | | | | | |
|  | |  |  |  |  | - I **refused** the request 🡪 forwarded to question D4 | | | | | | | | | | | | | | | | | | |
|  | |  |  |  |  | - **Decision-making** is **ongoing** | | | | | | | | | | | | | | | | | | |
|  | |  |  |  |  | - The patient **withdrew** the request | | | | | | | | | | | | | | | | | | |
|  | |  |  |  |  | - The patient **died before** I made the **decision** on the request | | | | | | | | | | | | | | | | | | |
|  | |  |  |  |  | - The patient **died** after I granted the request, but **before** I could **perform** the euthanasia | | | | | | | | | | | | | | | | | | |
|  | | In the past 5 years, have you ever **refused** an explicit request? | | | | - Yes | | | | | | | | | | | | | | | | | | |
|  | |  |  |  |  | - No 🡪 forwarded to **Section E** | | | | | | | | | | | | | | | | | | |
|  | | How long **before** the explicit request did you first discuss euthanasia with this patient? *(Enter the number of days, weeks, months* ***or*** *years; you may estimate if necessary)* | | | | days | | | | | | | | | | | | | | | | | | |
|  | |  |  |  |  | weeks | | | | | | | | | | | | | | | | | | |
|  | |  |  |  |  | months | | | | | | | | | | | | | | | | | | |
|  | |  |  |  |  | years | | | | | | | | | | | | | | | | | | |
|  | |  |  |  |  | - N/A, I did not discuss this with the patient prior to the explicit request | | | | | | | | | | | | | | | | | | |
|  | | How many conversations did you have with the patient about euthanasia **before the patient made the explicit request?** *(you may estimate if necessary)* | | | | conversations | | | | | | | | | | | | | | | | | | |
|  | |  |  |  |  | - None | | | | | | | | | | | | | | | | | | |
|  | | Did the patient have a written euthanasia will? | | | | - Yes | | | | | | | | | | | | | | | | | | |
|  | |  |  |  |  | - No | | | | | | | | | | | | | | | | | | |
|  | | **If dementia based on written euthanasia will,** was it sufficiently clear to you from the written will in which situation the patient wanted euthanasia to be performed? | | | | - Yes | | | | | | |  | | | | | | | | | | | |
|  | |  |  |  |  | - Somewhat | | | | | | |  | | | | | | | | | | | |
|  | |  |  |  |  | - No | | | | | | |  | | | | | | | | | | | |
|  | | **If dementia based on written euthanasia will,** what specific situations were described in the euthanasia will? | | | | | | | | | | | | | | | | | | | | | | |
|  | | **If dementia based on written euthanasia will,** can you explain how you interpreted these situations? | | | | | | | | | | | | | | | | | | | | | | |
|  | | **If dementia based on written euthanasia will,** can you briefly explain the preliminary process? (E.g. with whom did the patient discuss the euthanasia will, with whom did you discuss the request, topics of these discussions) | | | | | | | | | | | | | | | | | | | | | | |
|  | | **If dementia based on written euthanasia will,** the following questions concern the period when, according to those involved, the patient was in the situation described in the euthanasia declaration and you proceeded to perform the euthanasia. | | | | | | | | | | | | | | | | | | | | | | |
|  | | **If dementia based on written euthanasia will,** how long **before** the euthanasia was performed did the patient last update the written euthanasia will? *(Enter the number of days, weeks, months or years; you may estimate if necessary)* | | | | | | days | | | | | | | | | | | | | | | | |
|  | |  |  |  |  |  |  | weeks | | | | | | | | | | | | | | | | |
|  | |  |  |  |  |  |  | months | | | | | | | | | | | | | | | | |
|  | |  |  |  |  |  |  | years | | | | | | | | | | | | | | | | |
|  | |  |  |  |  |  |  | - Don’t know | | | | | | | | | | | | | | | | |
|  | |  |  |  |  |  |  | - Patient had not updated the written euthanasia will | | | | | | | | | | | | | | | | |
|  | | **If dementia based on written euthanasia will, after** the patient was, according to those involved, in the **situation** for which the euthanasia declaration was drawn up; | | | | | | **Yes** | | | | | | | | **No** | | | | | | **Don’t know** | | |
|  | | 1. Has the euthanasia will been discussed with relatives? | | | | | |  | | | | | | | |  | | | | | |  | | |
|  | | 1. Has the euthanasia will been discussed with the patient? | | | | | |  | | | | | | | |  | | | | | |  | | |
|  | | 1. Did the patient still support the request? | | | | | |  | | | | | | | |  | | | | | |  | | |
|  | | **Depending on the answer to question D12c,** what showed that the patient still/no longer supported the euthanasia request? Why do you doubt that the patient was still supportive of the euthanasia request? | | | | | | | | | | | | | | | | | | | | | | |
|  | | **If dementia based on written euthanasia will,** on whose initiative was the euthanasia will raised **after** the patient, according to those involved, was in the **situation** for which the euthanasia declaration was drawn up? | | | | | | - Patient | | | | | | | | | | | | | | | | |
|  | |  |  |  |  |  |  | - Relative(s) | | | | | | | | | | | | | | | | |
|  | |  |  |  |  |  |  | - Healthcare provider(s) | | | | | | | | | | | | | | | | |
|  | |  |  |  |  |  |  | - Other, **namely:** | | | | | | | | | | | | | | | | |
|  | | What was the patient's age **at the time** euthanasia **was performed**? | | | | | | | year | | | | | | | | | | | | | | | |
|  | | What was the gender of the patient? | | | | | | | - Male | | | | | | | | | | | - Female | | - Other | | |
|  | | Did the patient have a partner **at the time** euthanasia was performed? | | | | | | | - Yes, living together | | | | | | | | | | | | | | | |
|  | |  |  |  |  |  |  |  | - Yes, not living together | | | | | | | | | | | | | | | |
|  | |  |  |  |  |  |  |  | - No, widow/widower | | | | | | | | | | | | | | | |
|  | |  |  |  |  |  |  |  | - No, other | | | | | | | | | | | | | | | |
|  | | Where did the patient stay the **longest** in the last 3 months of his/her life? | | | | | | | - At home or with relatives at home | | | | | | | | | | | | | | | |
|  |  |  |  |  |  |  |  |  | - Hospital | | | | | | | | | | | | | | | |
|  |  |  |  |  |  |  |  |  | - Hospice | | | | | | | | | | | | | | | |
|  |  |  |  |  |  |  |  |  | - Nursing home or residential home | | | | | | | | | | | | | | | |
|  | |  |  |  |  |  |  |  | - Other, **namely**: | | | | | | | | | | | | | | | |
|  | | What was the main diagnosis? | | | | | | | | | | | | | | | | | | | | | | |
|  | | - N/A, there was no condition | | | | | | | | | | | | | | | | | | | | | | |
|  | | Which of the following situations applied to the patient?  *(one or more answers possible)* | | | | - Patient had cancer | | | | | | | | | | | | | | | | | | |
|  | |  |  |  |  | - Patient had another (life-threatening) physical condition | | | | | | | | | | | | | | | | | | |
|  | |  |  |  |  | - Patient had dementia | | | | | | | | | | | | | | | | | | |
|  | |  |  |  |  | - Patient had a psychiatric condition | | | | | | | | | | | | | | | | | | |
|  | |  |  |  |  | - Patient had an accumulation of health problems related to old age | | | | | | | | | | | | | | | | | | |
|  | |  |  |  |  | - Patient had psychosocial or existential problems | | | | | | | | | | | | | | | | | | |
|  | |  |  |  |  | - Other, **namely**: | | | | | | | | | | | | | | | | | | |
|  | | **If dementia,** what form of dementia was diagnosed? | | | | - Dementia not diagnosed by a doctor | | | | | | | | | | | | | | | | | | |
|  | |  |  |  |  | - Dementia unspecified | | | | | | | | | | | | | | | | | | |
|  | |  |  |  |  | - Alzheimer's dementia | | | | | | | | | | | | | | | | | | |
|  | |  |  |  |  | - Vascular dementia | | | | | | | | | | | | | | | | | | |
|  | |  |  |  |  | - Other, **namely**: | | | | | | | | | | | | | | | | | | |
|  | | **If dementia,** what stage of dementia was present **at the time** euthanasia was performed? | | | | - Early stage | | | | | | | | | | | | | | | | | | |
|  | |  |  |  |  | - Middle stage | | | | | | | | | | | | | | | | | | |
|  | |  |  |  |  | - Advanced | | | | | | | | | | | | | | | | | | |
|  | | **If dementia,** were there any additional conditions **at the time** euthanasia was performed? | | | | - Yes, **namely:** | | | | | | | | | | | | | | | | | | |
|  | |  |  |  |  | - No | | | | | | | | | | | | | | | | | | |
|  | | **If an accumulation of health problems related to old age,** which health problems related to old age did the patient have? *(one or more answers possible)* | | | | - Impaired vision | | | | | | | | - Balance problems | | | | | | | | | | |
|  | |  |  |  |  | - Impaired hearing | | | | | | | | - Cognitive decline | | | | | | | | | | |
|  | |  |  |  |  | - Osteoporosis | | | | | | | | - Other, **namely**: | | | | | | | | | | |
|  | |  |  |  |  | - Osteoarthritis | | | | | | | |  |  |  |  |  |  |  |  |  |  |  |
|  | | How long was the patient under your care **at the time of the** euthanasia **performance**? | | | | - Less than one month | | | | | | | | | | | | | | | | | | |
|  | |  |  |  |  | - 1 to 12 months | | | | | | | | | | | | | | | | | | |
|  | |  |  |  |  | - More than 12 months | | | | | | | | | | | | | | | | | | |
|  | | To what extent was the patient **dependent** on others **at the time** euthanasia was performed? | | | | - Almost independently | | | | | | | | | | | | | | | | | | |
|  | |  |  |  |  | - Limited care-dependent, e.g. needed some guidance from others | | | | | | | | | | | | | | | | | | |
|  | |  |  |  |  | - Highly dependent on others in daily life | | | | | | | | | | | | | | | | | | |
|  | | What was the treatment mainly focused on **at the time of the explicit request**? *(choose the most important)* | | | | - Curation | | | | | | | | | | - Palliation | | | | | | | | |
|  | |  |  |  |  | - Rehabilitation | | | | | | | | | | - N/A, patient did not receive treatment | | | | | | | | |
|  | |  |  |  |  | - Life prolonging | | | | | | | | | |  |  |  |  |  |  |  |  |  |
|  | | How did you estimate the patient's life expectancy **at the time of performing** euthanasia? | | | | - Less than 1 week | | | | | | | | | | - 6 to 12 months | | | | | | | | |
|  | |  |  |  |  | - 1 to 4 weeks | | | | | | | | | | - more than 12 months | | | | | | | | |
|  | |  |  |  |  | - 1 to 5 months | | | | | | | | |  | | | | | | | | | |
|  | | To what extent was substantive communication with the patient possible **at the time** euthanasia was performed? | | | | - Good | | | | | | | | | | - Moderate | | | | | | | | |
|  | |  |  |  |  | - Fair | | | | | | | | | | - Hardly to no | | | | | | | | |
|  | | What were the patient's **main reasons for explicitly requesting** euthanasia?  *(one or more answers possible)* | | | | | | | | | | | | | | | | | | | | | | |
|  | | - General weakness/fatigue | | | - Dependency | | | | | | | | | | | | | | | | | | | |
|  | | - Shortness of breath | | | - (Fear of) losing control of one's own life | | | | | | | | | | | | | | | | | | | |
|  | | - Pain | | | - Not wanting to be a burden to family/surroundings | | | | | | | | | | | | | | | | | | | |
|  | | - Other physical complaints | | | - No longer living independently | | | | | | | | | | | | | | | | | | | |
|  | | - Depressive feelings | | | - Suffering with no prospect of improvement | | | | | | | | | | | | | | | | | | | |
|  | | - Fear | | | - Having no purpose in life | | | | | | | | | | | | | | | | | | | |
|  | | - Cognitive decline | | | - Loss of dignity | | | | | | | | | | | | | | | | | | | |
|  | | - Physical deterioration | | | - Completed life/tired of living | | | | | | | | | | | | | | | | | | | |
|  | | - Disability/immobility | | | - Other, **namely**: | | | | | | | | | | | | | | | | | | | |
|  | | - Death of a loved one | | |  | | | | | | | | | | | | | | | | | | | |
|  | | - Loneliness | | |  |  |  |  |  |  |  |  |  |  |  |  |  |  |  |  |  |  |  |  |
|  | | Did it mainly relate to current suffering or fear of suffering in the future? | | | - Current suffering | | | | | | | | | | | | | | | | | | | |
|  | |  |  |  | - Fear of future suffering | | | | | | | | | | | | | | | | | | | |
|  | | **If dementia,** what did the patient's suffering consist of **at the time** euthanasia was performed? | | | | | | | | | | | | | | | | | | | | | | |
|  | | In your opinion, **at the time euthanasia was performed,** was there | | | | | | | | | | | No | | | | | | | To some extent | | | Yes | |
|  | | 1. a patient with decisional capacity? | | | | | | | | | | |  | | | | | | |  | | |  | |
|  | | 1. unbearable suffering? | | | | | | | | | | |  | | | | | | |  | | |  | |
|  | | 1. suffering without prospect of improvement? | | | | | | | | | | |  | | | | | | |  | | |  | |
|  | | 1. a voluntary and well-considered request? | | | | | | | | | | |  | | | | | | |  | | |  | |
|  | | 1. alternative treatment options? | | | | | | | | | | |  | | | | | | |  | | |  | |
|  | | **Dependent on answer question D33a-d:** In your opinion, why was there no or to some extent a patient with decisional capacity/ unbearable suffering suffering without prospect of improvement/ a voluntary and well-considered request? | | |  | | | | | | | | | | | | | | | | | | | |
|  | | **Depending on answer question D33e:** In your opinion, why were there (to some extent) alternative treatment options? | | |  | | | | | | | | | | | | | | | | | | | |
|  | | Did you consult another physician **after the explicit euthanasia request**?  *(one or more answers possible)* | | | - No | | | | | | | | | | | | | | | | | | | |
|  | |  |  |  | - Yes, a SCEN physician | | | | | | | | | | | | | | | | | | | |
|  | |  |  |  | - Yes, a psychiatrist | | | | | | | | | | | | | | | | | | | |
|  | |  |  |  | - Yes, another doctor, **namely**: | | | | | | | | | | | | | | | | | | | |
|  | | What were the opinions of close relatives on the explicit euthanasia request? | | | - N/A, no relatives were involved | | | | | | | | | | | | | | | | | | | |
|  | |  |  |  | - They had a neutral point of view | | | | | | | | | | | | | | | | | | | |
|  | |  |  |  | - They supported the patient's request | | | | | | | | | | | | | | | | | | | |
|  | |  |  |  | - They **did not** support the patient's request | | | | | | | | | | | | | | | | | | | |
|  | |  |  |  | - They were divided | | | | | | | | | | | | | | | | | | | |
|  | |  |  |  | - Don't know | | | | | | | | | | | | | | | | | | | |
|  | | Can you indicate whether you have had any experience in this with the following aspects of euthanasia? | | | | | | | | | | | | | | | | | **Yes** | | | **No** | | |
|  | | 1. Assessing whether the due care criteria could be met | | | | | | | | | | | | | | | | |  | | |  | | |
|  | | 1. The time taken by the decision-making process | | | | | | | | | | | | | | | | |  | | |  | | |
|  | | 1. The emotional burden of the preperation | | | | | | | | | | | | | | | | |  | | |  | | |
|  | | 1. Administration of lethal medication | | | | | | | | | | | | | | | | |  | | |  | | |
|  | | 1. The emotional burden of the performance | | | | | | | | | | | | | | | | |  | | |  | | |
|  | | 1. The administrative burden of reporting | | | | | | | | | | | | | | | | |  | | |  | | |
|  | | 1. Waiting for the verdict of the euthanasia review committee | | | | | | | | | | | | | | | | |  | | |  | | |
|  | | 1. Other healthcare providers' reactions | | | | | | | | | | | | | | | | |  | | |  | | |
|  | | 1. Dealing with the patient's relatives | | | | | | | | | | | | | | | | |  | | |  | | |
|  | | 1. Were there any other issues you encountered? **If yes**, please explain below: | | | | | | | | | | | | | | | | | | | | | | |
|  | |  |  |  |  |  |  |  |  |  |  |  |  |  |  |  |  |  |  |  |  |  |  |  |
|  | | In this case study, have you had the experiences below? | | | | | | | | | | | | | | | | | | | Yes | | | No |
|  | | 1. I was under the impression that the patient had made the euthanasia request under **pressure from relatives** | | | | | | | | | | | | | | | | | | |  | | |  |
|  | | 1. I felt pressured **by the patient** to decide on euthanasia | | | | | | | | | | | | | | | | | | |  | | |  |
|  | | 1. I felt pressured **by relatives** of the patient to decide on euthanasia | | | | | | | | | | | | | | | | | | |  | | |  |
|  | | 1. I felt pressured **by relatives** of the patient to **refuse** the euthanasia request | | | | | | | | | | | | | | | | | | |  | | |  |
|  | | 1. I felt pressured by the patient or their relatives to perform euthanasia **as soon as possible** | | | | | | | | | | | | | | | | | | |  | | |  |
|  | | How long did the decision-making on this euthanasia request take **from the explicit request until you performed the euthanasia**? *(Enter the number of days, weeks,* ***or*** *months)* | | | | | | days | | | | | | | | | | | | | | | | |
|  | |  |  |  |  |  |  | weeks | | | | | | | | | | | | | | | | |
|  | |  |  |  |  |  |  | months | | | | | | | | | | | | | | | | |
|  | | Have you performed euthanasia or physician-assisted suicide? | | | | | | - Performed euthanasia | | | | | | | | | | | | | | | | |
|  | |  |  |  |  |  |  | - Assisted in suicide | | | | | | | | | | | | | | | | |
|  | | **If dementia,** did you administer premedication* when performing the euthanasia? *(* Medication to put the patient into a light sleep state and prevent any pain or fright reactions, e.g. midazolam).* | | | | | | - Yes | | | | | | | | | | | | | | | | |
|  | |  |  |  |  |  |  | - No | | | | | | | | | | | | | | | | |
|  | | Where did the euthanasia or physician-assisted suicide take place? | | | | | | - At home or with relatives at home | | | | | | | | | | | | | | | | |
|  | |  |  |  |  |  |  | - Hospital | | | | | | | | | | | | | | | | |
|  | |  |  |  |  |  |  | - Hospice | | | | | | | | | | | | | | | | |
|  | |  |  |  |  |  |  | - Nursing home or residential home | | | | | | | | | | | | | | | | |
|  | |  |  |  |  |  |  | - Anders, **nl**: | | | | | | | | | | | | | | | | |
|  | | 1. Did complications or unexpected events occur during the performance of euthanasia or assistance in suicide? | | | | | | - Yes | | | | | | | | | | | | | | | | |
|  | |  |  |  |  |  |  | - No 🡪 forwarded to **question D45** | | | | | | | | | | | | | | | | |
|  | | 1. **If yes,** what complications or unexpected events occurred during the performance of euthanasia or assistance in suicide? | | | | | | | | | | | | | | | | | | | | | | |
|  | |  | | | | | | | | | | | | | | | | | | | | | | |
|  | | 1. Have you reported this case to an euthanasia review committee? | | | | | | - Yes 🡪 forwarded to **question D46** | | | | | | | | | | | | | | | | |
|  | |  |  |  |  |  |  | - No | | | | | | | | | | | | | | | | |
|  | | 1. **If no,** why did you not report this case to an euthanasia review committee? | | | | | | | | | | | | | | | | | | | | | | |
|  | | How did you perceive the role of the municipal coroner? *(one or more answers possible)* | | | | | | - As important for completion of the termination of life | | | | | | | | | | | | | | | | |
|  | |  |  |  |  |  |  | - As an aid to legal settlement | | | | | | | | | | | | | | | | |
|  | |  |  |  |  |  |  | - As a check on my actions | | | | | | | | | | | | | | | | |
|  | |  |  |  |  |  |  | - As an intrusion into the intimacy of death | | | | | | | | | | | | | | | | |
|  | |  |  |  |  |  |  | - Other, **namely**: | | | | | | | | | | | | | | | | |
|  | | 1. Did the regional review committee ask you for additional information about this case during the review procedure? | | | | | | - Yes | | | | | | | | | | | | | | | | |
|  | |  |  |  |  |  |  | - No🡪 forwarded to **question D49** | | | | | | | | | | | | | | | | |
|  | | 1. **If yes**, can you briefly describe what information the review committee asked for? | | | | | | | | | | | | | | | | | | | | | | |
|  | | How did you respond to the request for additional information? *(one or more answers possible)* | | | | | - By letter | | | | | | | | | | | | - In a personal conversation | | | | | |
|  | |  |  |  |  |  | - By e-mail | | | | | | | | | | | | - Other, **namely:** | | | | | |
|  | |  |  |  |  |  | - By phone | | | | | | | | | | | |  |  |  |  |  |  |
|  | | What did you think of the letter informing you of the review committee's judgement? *(one or more answers possible)* | | | | | - Good | | | | | | | | | | | | - Insensitive | | | | | |
|  | |  |  |  |  |  | - Informative | | | | | | | | | | | | - Unclear | | | | | |
|  | |  |  |  |  |  | - Standard text | | | | | | | | | | | | - Other, **namely:** | | | | | |
|  | |  |  |  |  |  | - Formal | | | | | | | | | | | |  |  |  |  |  |  |
|  | | In general, how did you experience the whole procedure surrounding this report? *(one or more answers possible)* | | | | | - Neutral | | | | | | | | | | | | - Supportive | | | | | |
|  | |  |  |  |  |  | - Relieving | | | | | | | | | | | | - Incriminating | | | | | |
|  | |  |  |  |  |  | - Insightful | | | | | | | | | | | | - Burdensome | | | | | |
|  | |  |  |  |  |  | - Time-consuming | | | | | | | | | | | | - Other, **namely:** | | | | | |
|  | |  |  |  |  |  | - Breach of privacy | | | | | | | | | | | |  |  |  |  |  |  |
|  | | 1. Did you have an aftercare conversation about the euthanasia or physician-assisted suicide with the relatives? | | | | | - Yes, once | | | | | | | | | | | | | | | | | |
|  | |  |  |  |  |  | - Yes, multiple times | | | | | | | | | | | | | | | | | |
|  | |  | | | | | - No 🡪 forwarded to **Section E** | | | | | | | | | | | | | | | | | |
|  | | 1. **If yes,** what was discussed in this follow-up conversation(s)? | | | | |  | | | | | | | | | | | | | | | | | |
|  | | 1. **If yes**, did this lead to the provision of care? | | | | | - No | | | | | | | | | | | | | | | | | |
|  | |  |  |  |  |  | - Yes, **namely**: | | | | | | | | | | | | | | | | | |
| If the doctor has indicated refusal of the request in question D2 or D3, questions 41 to 51 are replaced by questions 52 to 57 | | | | | | | | | | | | | | | | | | | | | | | | |
|  | | What was the **main** reason for refusing the request? | | | | | - I never perform euthanasia | | | | | | | | | | | | | | | | | |
|  | |  |  |  |  |  | - Objections from family | | | | | | | | | | | | | | | | | |
|  | |  |  |  |  |  | - Possible failure to meet due care criteria, **namely:** | | | | | | | | | | | | | | | | | |
|  | |  |  |  |  |  | - Personal objections specific to this case, **namely:** | | | | | | | | | | | | | | | | | |
|  | |  |  |  |  |  | - Other, **namely:** | | | | | | | | | | | | | | | | | |
|  | | In this case, do you think the euthanasia would have been judged as careful by the review board **if you did perform it?** | | | | | - Yes | | | | | | | | | | | | | | | | | |
|  | |  |  |  |  |  | - No | | | | | | | | | | | | | | | | | |
|  | |  |  |  |  |  | - Don't know | | | | | | | | | | | | | | | | | |
|  | | What decisions regarding **treatment** and care were made **after refusing** the euthanasia request? *(one or more answers possible)* | | - No change in treatment and care | | | | | | | | | | | | | | - Palliative sedation | | | | | | |
|  | |  |  | - Curative or life-prolonging treatment stopped | | | | | | | | | | | | | | - Transfer to hospice | | | | | | |
|  | |  |  | - Symptom management aimed at palliation started/ intensified | | | | | | | | | | | | | | - Transfer to rehabilitation place | | | | | | |
|  | |  |  | - Treatment restriction agreed: e.g. no treatment in case of infection | | | | | | | | | | | | | | - Transfer to other care facility | | | | | | |
|  | |  |  | - Psychological counselling started/ intensified | | | | | | | | | | | | | | - Discharge to home | | | | | | |
|  | |  |  | - Expanding social activities | | | | | | | | | | | | | | - Home care was started or expanded | | | | | | |
|  | |  |  | - Expanding care | | | | | | | | | | | | | | - Anders, **nl:** | | | | | | |
|  | | 1. Did you **refer** the patient **after refusing the euthanasia request**? | | - Yes, to Euthanasia Expertise Centre 🡪 forwarded to **question 56** | | | | | | | | | | | | | | | | | | | | |
|  | |  |  | - Yes, to another physician 🡪 forwarded to **question 56** | | | | | | | | | | | | | | | | | | | | |
|  | |  |  | - No | | | | | | | | | | | | | | | | | | | | |
|  | | 1. **If no**, did the patient himself seek another physician who was willing to grant his/her request? | | - Yes, through the Euthanasia Expertise Centre 🡪 forwarded to **question 56** | | | | | | | | | | | | | | | | | | | | |
|  | |  |  | - Yes, another doctor 🡪 forwarded to **question 56** | | | | | | | | | | | | | | | | | | | | |
|  | |  |  | - Don't know 🡪 forwarded to **question 56** | | | | | | | | | | | | | | | | | | | | |
|  | |  |  | - No | | | | | | | | | | | | | | | | | | | | |
|  | | 1. I**f no**, what do you think was the reason for **not** seeking another doctor? | | | | | | | | | | | | | | | | | | | | | | |
|  | | 1. Did you ever **discuss** euthanasia with the patient **after refusing his/her request**? *(one or more answers possible)* | | - Yes, on my initiative | | | | | | | | | | | | | | | | | | | | |
|  | |  |  | - Yes, at the patient's initiative | | | | | | | | | | | | | | | | | | | | |
|  | |  |  | - No 🡪 forwarded to **question 57** | | | | | | | | | | | | | | | | | | | | |
|  | | 1. **If yes**, were any agreements made as a result of that conversation? | | - Yes | | | | | | | | | | | | | | | | | | | | |
|  | |  |  | - No 🡪 forwarded to **question 57** | | | | | | | | | | | | | | | | | | | | |
|  | | 1. **If yes,** what was agreed upon? | | | | | | | | | | | | | | | | | | | | | | |
|  | | Did the patient die? | | - No | | | | | | | | | | | | | - Yes, by euthanasia performed by another physician | | | | | | | |
|  | |  |  | - Don't know | | | | | | | | | | | | | - Yes, by stopping eating and drinking | | | | | | | |
|  | |  |  | - Yes, naturally | | | | | | | | | | | | | - Yes, through ingestion of self-administered lethal drugs | | | | | | | |
|  | |  |  | - Yes, by euthanasia performed by the Euthanasia Expertise Centre | | | | | | | | | | | | | - Yes, by suicide | | | | | | | |

| **E. Statements** | | | | | | | | |
| --- | --- | --- | --- | --- | --- | --- | --- | --- |
| Please indicate the extent to which you agree or disagree with the following statements. These are your opinions and not what is legally permissible. | | Totally agree | Agree | | Neither agree/disagree | Disagree | | Totally disagree |
|  | I have sufficient knowledge about the content of the euthanasia law. |  |  | |  |  | |  |
|  | I am sufficiently competent in providing palliative care. |  |  | |  |  | |  |
|  | Good palliative care makes euthanasia unnecessary. |  |  | |  |  | |  |
|  | Everyone has the right to decide their own life and death. |  |  | |  |  | |  |
|  | In the case of a euthanasia will signed by a patient with dementia when they still had decisional capacity, it is impossible to determine **when** this request should be granted. |  |  | |  |  | |  |
|  | It is **impossible to assess** whether a patient with dementia without decisional capacity is suffering unbearably and hopelessly. |  |  | |  |  | |  |
|  | A patient with dementia may suffer unbearably from dementia. |  |  | |  |  | |  |
|  | Dying by stopping eating and drinking can be a good alternative to euthanasia. |  |  | |  |  | |  |
|  | If morphine is titrated on pain guidance, it can hasten the end of life. |  |  | |  |  | |  |
|  | Administration of high doses of morphine to hasten the end of life of a severely suffering patient at their request can be a good way to perform euthanasia. |  |  | |  |  | |  |
|  | I experience pressure from society to grant euthanasia requests. |  |  | |  |  | |  |
|  | I want to know the opinions of close relatives on a euthanasia request. |  |  | |  |  | |  |
|  | I take into account the opinions of close relatives when deciding on a euthanasia request |  |  | |  |  | |  |
|  | Since the start of the Expertise Centre, I am more likely to refuse euthanasia requests |  |  | |  |  | |  |
|  | I think it is right that only a doctor should be allowed to perform euthanasia. |  |  | |  |  | |  |
|  | There should be more legal options for citizens to provide assisted suicide in cases of tired of living. |  |  | |  |  | |  |
|  | I prefer euthanasia (compared to physician-assisted suicide) because then I can be sure the execution will go well. |  |  | |  |  | |  |
|  | I prefer to physician-assisted suicide (over euthanasia) because then I am sure the patient wants it. |  |  | |  |  | |  |
|  | There are situations where I would provide to physician-assisted suicide but would not perform euthanasia. |  |  | |  |  | |  |
|  | Do you have a **preference** for euthanasia over physician-assisted suicide, or vice versa, in the following patient groups? | **Euthanasia** | | **Physician-assisted suicide** | | | **No preference** | |
|  | Life-threatening condition |  | |  | | |  | |
|  | Dementia |  | |  | | |  | |
|  | Psychiatric suffering |  | |  | | |  | |
|  | Accumulation of health problems related to old age |  | |  | | |  | |
|  | Tired of living |  | |  | | |  | |
|  | Would you like to explain your choices on the previous question? | | | | | | | |

| **F. Closure** | | | | | | |
| --- | --- | --- | --- | --- | --- | --- |
|  | 1. What is your gender? | Your gender: | | - Male | - Female | - Other |
|  | 1. What is your age? | Your age: | | years | | |
|  | 1. Do you have a religion or? | - No | | | | |
|  |  | - Yes, **namely**: | | | | |
|  | How many patients under your care died in the past year? (please estimate | patients | | | | |
|  | Have you had any additional training (in addition to your regular training) in palliative care? | - No | | | | |
|  |  | - Yes, certified palliative care training | | | | |
|  |  | - Yes, other, **namely**: | | | | |
|  |  |  | | | | |
|  | 1. Are you familiar with the Euthanasia Code of Practice of the Regional Euthanasia Review Committee? | - Yes | - No 🡪 forwarded to **In conclusion** | | | |
|  | 1. **If yes,** how often do you consult the Euthanasia Code? | - In any euthanasia request | | | | |
|  |  | - In some euthanasia requests | | | | |
|  |  | - Never | | | | |
|  | 1. **If yes**, do you think the Euthanasia Code contains useful information? | - Yes, definitely | | | | |
|  |  | - Yes, somewhat | | | | |
|  |  | - No | | | | |

**In conclusion**

We will do a number of in-depth interviews in which there will be enough time to discuss **your own experiences of euthanasia**. We would like to ask you if you would like to participate in such an interview. If you are willing to do so, we ask you to provide your name and phone number or your e-mail address below. **This will break anonymity, but data will remain confidential.** We will eventually approach selected doctors for an interview.

|  | May we approach you for an interview? | - Yes | - No 🡪 forwarded to Comments |
| --- | --- | --- | --- |
|  | What is your name and phone number? |  | |
|  | What is your e-mail address? |  | |

Comments:

**Thank you very much for completing the questionnaire.**

Don't forget to submit your answers by clicking 'next'!

If you have any **questions** or **comments** regarding this study or this questionnaire, please contact:
