# Supplementary material for: Physicians’ views on the role of relatives in euthanasia and physician-assisted suicide decision-making: a mixed-methods study among physicians in the Netherlands
Source: BMC Med Ethics. 2024 Apr 5;25:43. doi: 10.1186/s12910-024-01031-1 (PMC10996154; doi:10.1186/s12910-024-01031-1)
Supplement: Supplementary file 2 — Supplementary Material 2. [file 12910_2024_1031_MOESM2_ESM.docx]

**Additional file 2 – topic list interviews**

Interviews round 1 (in bold the topic that is relevant for the current study)

- Conceivability of euthanasia
  - Somatic vs. non-somatic conditions
  - Reasons to find performing euthanasia conceivable or not
  - Changes in conceivability over time
- Communication with patients about a euthanasia request in case of refusal of the request
- Experiences with the Euthanasia Expertise Center
  - Referral to the Euthanasia Expertise Center
  - Reactions from patients and relatives to referral
- Views on euthanasia in case of “tired of living”
- Experiences with pressure
  - Pressure from patients, relatives and colleagues
  - Pressure from society in general
- **Views on the role of relatives in EAS decision-making**
  - **Importance of relatives’ opinion**
  - **Experience and dealing with opposing views**
- Preferences for euthanasia and/or physician-assisted suicide
  - Reasons for a certain preference
  - Situation-dependency
  - Changes in preference over time
- Experiences with review by the Dutch Regional Euthanasia Review Committees
  - Case description
  - Role of the inspection and general prosecutor
  - Views on usefulness of the review system

Interviews round 2

- Importance of knowing relatives’ opinion
- Extent to which relatives’ opinion is taken into account
- Dealing with relatives’ opinion in practice [including potential opposing views]
- Relationship with perceived pressure
