# Supplementary material for: Physicians’ views on the role of relatives in euthanasia and physician-assisted suicide decision-making: a mixed-methods study among physicians in the Netherlands
Source: BMC Med Ethics. 2024 Apr 5;25:43. doi: 10.1186/s12910-024-01031-1 (PMC10996154; doi:10.1186/s12910-024-01031-1)
Supplement: Supplementary file 3 — Supplementary Material 3. [file 12910_2024_1031_MOESM3_ESM.docx]

**Additional file 3 – logistic regression analysis agree versus neutral category**

| **Table A1.** Among those physicians that would like to know close relatives’ opinion, characteristics of physicians who take close relatives’ opinion into account (agree vs. neutral) (n=386) | | | | | |
| --- | --- | --- | --- | --- | --- |
|  | |  | **Agree**  **N=** | **Univariable** | **Multivariable** |
|  | |  | Row % | OR (95% CI) | OR (95% CI) |
| **DEMOGRAPHICS** | | | | | |
| **Gender^a^** | | |  |  |  |
|  | Male (n=170) | | 51.2% | 1.00 |  |
|  | Female (n=215) | | 53.0% | 1.08 (0.72-1.61) |  |
| **Age (years)** | | |  | 1.01 (0.98-1.03) |  |
| **Religious belief** | | |  |  |  |
|  | No (n=264) | | 51.5% | 1.00 |  |
|  | Yes (n=119 | | 52.9% | 1.06 (0.69-1.63) |  |
| **PROFESSIONAL CHARACTERISTICS** | | | | | |
| **Specialty** | | |  |  |  |
|  | General practitioner (n=187) | | 45.5% | 1.00 | 1.00 |
|  | Clinical specialist (n=116) | | 61.2% | **1.89 (1.18-3.03)** | **1.89 (1.18-3.03)** |
|  | Elderly care physician (n=81) | | 54.3% | 1.43 (0.85-2.41) | 1.43 (0.85-2.41) |
| **Years of working experience** | | | | 1.01 (0.99-1.03) |  |
| **Consultant palliative care/member palliative care team** | | | | | |
|  | No (n=360) | | 52.5% | 1.00 |  |
|  | Yes (n=25) | | 52.0% | 0.98 (0.44-2.21) |  |
| **SCEN physician** | | |  |  |  |
|  | No (n=375) | | 52.0% | 1.00 |  |
|  | Yes (n=10) | | 70.0% | 2.15 (0.55-8.46) |  |
| **Ever received an explicit EAS request** | | | | | |
|  | No (n=88) | | 52.3% | 1.00 |  |
|  | Yes, but never performed EAS (n=97) | | 51.5% | 0.97 (0.55-1.73) |  |
|  | Yes, and ever performed EAS (n=199) | | 53.3% | 1.04 (0.63-1.72) |  |
| a There is one physician who indicated to have gender ‘other’. This is treated as a missing value in this analyses due to problems with statistical power.  Missing values: gender 1, age 2, religious belief 3, specialty 2, years of working experience 3, consultant palliative care/member of palliative care team 1, SCEN physician 1, ever received an explicit EAS request 2 | | | | | |
